# Digital Well-Being Training With Health Care Professionals: A Randomized Clinical Trial

**DOI:** 10.1001/jamainternmed.2025.3888

**Published:** 2025-08-18

**Authors:** Matthew J. Hirshberg, Richard J. Davidson, Luciana B. Velarde Arrisueño, José Miguel Olvera Puentes, Ximena Medina Bardalez, Byron S. Gonzalez, Simon B. Goldberg, Leandro I. Chernicoff

**Affiliations:** 1 Center for Healthy Minds, University of Wisconsin-Madison, Madison; 2 Department of Psychology, University of Wisconsin-Madison, Madison; 3 AtentaMente Consultores A.C., Mexico City, Mexico; 4 Harvard College; 5 Department of Counseling Psychology, University of Wisconsin-Madison, Madison; 6 Department of Psychiatry, University of Wisconsin-Madison, Madison,

## Abstract

**Importance:**

High levels of health care professional (HCP) distress portend negative consequences for HCPs and health systems.

**Objective:**

To evaluate whether a 13-week digital well-being training could improve Mexican HCP distress and well-being.

**Design, Setting, and Participants:**

This 2-arm randomized clinical trial was conducted between March 2023 and March 2024 with 13-week postintervention and 37-week follow-up end points. Participants included 2315 adults with no previous well-being training experience who worked in 762 participating health care facilities across 7 Mexican states. Participants were randomly assigned (1:1) using stratified permuted blocks to well-being training or wait-list control. Outcomes assessors and study data analyst were masked to condition assignment. Assessments and intervention were conducted remotely.

**Intervention:**

The digital well-being training consisted of nine 2-hour zoom sessions and 13 weeks of the Healthy Minds Program smartphone app structured on the premise that well-being skills can be strengthened through psychoeducation and contemplative practice.

**Main Outcomes:**

The primary prespecified outcomes were participant-reported distress (aggregate of *z*-scored National Institutes of Health Toolbox perceived stress, and Perceived Stress and Patient Reported Outcomes Measurement Information System anxiety and depression scales; mean = 0, SD = 1) and the World Health Organization–5 measure of well-being (range 0-25).

**Results:**

Among 2315 participants, 1693 (73.1%) identified as female; 778 (33.4%) nurses, 736 (31.7%) physicians, 187 (8.1%) psychologists, 183 (7.9%) administrators, 154 (6.7%) dentists, 75 (3.2%) social workers, 64 (2.8%) nutritionists, 55 (2.4%) laboratory technicians, 40 (1.7%) physical therapists, 29 (1.3%) health promoters, and 8 (<0.1%) other health professionals; the mean (SD) age was 34.7 (11.4) years. Relative to control, in intention-to-treat analyses the intervention group reported 13-week reductions in distress (adjusted standardized mean difference [SMD], −0.24; 95% CI, −0.32 to −0.16) and increases in well-being (adjusted SMD, 0.27; 95% CI, 0.18 to 0.35) that increased in magnitude at 37-week follow-up (distress, −0.36; 95% CI, −0.44 to −0.28; well-being, 0.42; 95% CI, 0.34 to 0.50). Primary outcomes were robust in sensitivity analyses. There were 10 adverse events (intervention: 0.05% [6 of 1157]; control: 0.04% [4 of 1158]).

**Conclusions and Relevance:**

This randomized clinical trial found that scalable digital well-being training modestly improved Mexican HCP distress and well-being compared with a wait-list condition. As HCPs are essential to public health and report high levels of distress that interfere with their ability to provide optimal care, well-being training may be an avenue to better supporting HCPs.

**Trial Registration:**

ClinicalTrials.gov Identifier: NCT05767970.

## Key Points

**Question:**

Can a digital well-being training implemented at scale effectively reduce Mexican health care professional distress and increase well-being?

**Findings:**

This randomized clinical trial of 2315 health care professionals across 7 Mexican states found that participants assigned to digital well-being training reported statistically significant and clinically meaningful reductions in distress and increases in well-being that persisted at least 37 weeks postrandomization.

**Meaning:**

These findings suggest that it is possible to modestly but meaningfully improve health care professional distress and well-being at scale, with potential benefits for patient care and health systems that require further study.

## Teaser

This randomized clinical trial examines whether a 13- week digital well-being training could improve well-being and reduce stress among health care professionals in Mexico.

## Introduction

Health care professionals (HCPs) across the world report high rates of distress and burnout, with especially high burnout rates in low- and middle-income countries.^1,2,3^ Mexican HCPs face added difficulties such as long work hours, low pay, and staff shortages.^4^ For example, although the Organisation for Economic Co-ordination and Development recommends 3.2 physicians and 8.8 nurses per 100 000 people, Mexico had only 1.95 physicians and 2.85 nurses per 100 000 people in 2021.^5^ Elevated HCP distress is associated with poorer patient care and outcomes, threatening to undermine public health gains achieved by Mexico over the last decades.^6,7^

While growing awareness of HCP distress has led to calls to promote HCP well-being,^3,8^ there are few adequately powered randomized trials of interventions to reduce distress among HCPs, and none, to our knowledge, in low- and middle income countries.^9,10,11^ A 2023 systematic review identified only 7 RCTs (all with n ≤135) on HCP workplace well-being trainings.^9^ More recent trials of mindfulness and an app-based exercise intervention with medical students in Norway (n = 288) and Canadian HCPs (n = 288), respectively, reported reduced distress or depression and improved well-being relative to wait-list. However, neither of these studies included a follow-up.

Digital health interventions may be a promising approach to reducing HCP distress. Mexican HCPs report being reluctant to seek help due to concerns over disclosure, stigma, and documentation in medical records, which could impact professional licensing.^12^ Digital technologies have received positive attention due to their low marginal costs and potential for increased accessibility and anonymity.^13^ A 13-week digital well-being training consisting of 9 video call–based sessions and 13 weeks of a smartphone app demonstrated acceptability and potential benefits on depression and well-being in single-arm, open-label program improvement evaluations involving 1013 Mexican HCPs. These data, however, are subject to several potential biases. We sought to formally test whether this program improved distress and well-being among Mexican HCPs.

## Methods

### Trial Design and Participants

We conducted a 2-arm parallel RCT in Campeche, Coahuila, Nuevo León, Oaxaca, Querétaro, Sonora, and Jalisco states. Adults who worked as patient-facing HCPs or, in response to partners, as regional or state health care administrators in these states were eligible to enroll. Individuals already receiving mental health care remained eligible. Individuals were ineligible if they had previously attended a training by AtentaMente, the Mexican nonprofit organization that helped develop the intervention.

Participants were recruited at Secretary of Health system facilities through information sessions and health systems communications. Participants who prescreened as eligible in our Research Electronic Data Capture (REDCap) project received an autogenerated enrollment email invitation. Participants who consented to be individually randomly assigned to intervention or wait-list control and completed the baseline survey entered the randomization pool.

As required by partnering health systems, we obtained ethics board approval from the following states and institutions: Queretaro; Campeche; Jalisco (2 boards); Nuevo León; and the University of Wisconsin–Madison institutional review board. We registered the trial at ClinicalTrials.gov (NCT05767970). The trial protocol is in Supplement 1. This study followed the Consolidated Standards of Reporting Trials (CONSORT) reporting guideline.

### Randomization

Following completion of the baseline survey, on May 23, 2023, REDCap randomly assigned participants 1:1 to intervention or wait-list control using a permuted block assignment list stratified on state and health care facility resource tier created with the Carat package in R version 4.4.0.^14,15^ Participants were notified that if assigned to the intervention, participating in synchronous sessions with camera on may disclose their identity. All participant-reported outcomes were collected remotely through REDCap (ie, no assessor). The study data analyst was masked to condition assignment until analyses were completed.

### Intervention

The Integrated Stress Toolbox for Healthcare Providers is a 13-week intervention consisting of 8 weekly 2-hour video call sessions with a final session at week 13, and 13 weeks of companion content through the Healthy Mind Program app.^16,17^ To accommodate HCP schedules, all sessions are recorded and can be watched asynchronously. The intervention is based on the theory that well-being skills are trainable and promote well-being.^18^ Information about well-being was provided through lectures and podcast style “learns” on the app. Skills strengthening occurred through opportunities to apply this learning through cognitive-behavioral and contemplative techniques.^18^ For example, participants learned strategies to identify and reappraise cognitive distortions, clarify values, and practice mindfulness of breath, appreciation, compassion, and self-inquiry.^18,19^ The app allowed participants to reinforce learning by selecting the type and duration of practice.

The intervention was implemented in 4 cohorts of up to 500 participants that began and ended on the same weeks, were led by the same 2 instructors—one of whom was a practicing surgeon in Mexico—and were supported by 10 facilitators. Instructors had completed AtentaMente’s 1.5-year core certification training and had more than 1 year of teaching experience. At a minimum, facilitators had completed AtentaMente’s 2-week facilitator training. An external auditor observed all sessions and graded fidelity based on concordance with planned session content.

### Measures and Outcome

The study involved 8 assessments: baseline prior to random assignment, and 1-, 3-, 5-, 8-, 13-, 25-, and 37-week postrandomization assessments. The 13-week postintervention and 37-week follow-up were the preregistered primary end points. To reduce participant burden, only primary outcomes were assessed at all time points (eTable 1 in Supplement 2). The primary psychological distress outcome was the aggregate of *z*-scored 10-item Perceived Stress and Patient Reported Outcomes Measurement Information System (PROMIS) 8-item Adult Anxiety and Depression scales.^20,21^ We used a distress composite because a single distress factor may underly all forms of psychopathology and clinical diagnoses of anxiety and depression are highly cormorbid.^16,22,23^ However, because minimal clinically important difference (MCID) thresholds of at least 0.30 SD exist for anxiety and depression measures and help inform the clinical importance of change, in exploratory analyses we report on anxiety and depression outcomes disaggregated. The second primary outcome was well-being, assessed with the 5-item World Health Organization-5.^24^

Secondary outcomes included the 17-item Healthy Minds Index (ie, awareness, connection, insight, and purpose skill subscales),^25^ the 6-item Gratitude Questionnaire-6,^26^ and the Maslach Burnout Inventory Emotional Exhaustion (9-items) and Personal Accomplishment at Work (7-items) subscales.^27^ Depersonalization burnout was not assessed because of poor psychometrics in pilot data. Intervention adherence was communicated to participants as attending and/or watching at least 7 of 9 sessions and 300 minutes or longer of app use during the intervention.

The trial registration included tertiary participant-reported outcomes, patient satisfaction reports, dried blood spot assays and administrative records (eg, absenteeism). Tertiary outcomes, blood spots, and administrative records will be reported elsewhere. We were unable to collect reliable patient satisfaction reports. All study materials were in Spanish. All measures demonstrated adequate psychometrics with Mexican HCPs.

### Statistical Analysis

A priori minimum detectable effect size analyses were based on the maximum allowable sample of 4000 HCPs. These analyses indicated that, assuming 80% power, an intraclass correlation coefficient of 0.50, and statistical significance set to a Bonferroni-corrected 2-sided *P* < .006, the minimum detectable group by time interaction effect for primary outcomes would be a standardized mean difference (SMD) ≥ 0.04 and for secondary outcomes, SMD ≥ 0.05.

Statistical analyses and data cleaning processes were performed using R statistical software^15^ version 4.4.0 and MPlus (eMethods 1 in Supplement 2). As prespecified, we checked data quality by examining patterns of careless responding (eMethods 2 in Supplement 2). After observing no evidence of careless response patterns,^28^ all data were retained. Prespecified statistical significance was set to a 2-sided false discovery rate (FDR)–corrected *P* < .05 across all inferential tests.^29^

We first visually examined group change over time by plotting loess regressions of each outcome (eFigures 1 and 2 Supplement 2).^30^ For primary outcomes, we quantitatively compared mixed effects linear, loglinear, polynomial and piecewise linear change. In piecewise models, baseline to postintervention and postintervention to 37-week follow-up splines were each modeled as participant-level random intercepts and slopes. Piecewise models were the most parsimonious while avoiding overfitting. Piecewise models for burnout outcomes modeled the baseline to postintervention spline as a participant-level random intercept because burnout was assessed only at baseline and postintervention (eFigure 2 in Supplement 2). For secondary outcomes not assessed at follow-up, we estimated linear mixed-effects models with participant-level random intercepts and slopes. As prespecified, gender and age were included as covariates in all models. We followed the intention-to-treat principle, including all randomly assigned participants. Adjusted SMDs were estimated using the Feingold method.^31^

Group rates of missingness differed by approximately 6% at 13- and 37-week follow-ups, suggesting missing at random assumptions may have been violated. Consequently, we conducted prespecified pattern-mixture model sensitivity analyses.^32^ We multiply imputed 50 complete datasets through multivariate imputation by chained equations maintaining compatibility with the hierarchal structure of the data and modeling approach.^33^ Imputed values were scaled to 10% and 20% worse than imputed. Models were reestimated on each imputed, scaled dataset and results were pooled.^34^

In prespecified exploratory analyses, we tested treatment effect moderation by participant gender, age, employment category, and state with a 3-way interaction term between the moderating variable, group, and spline(s). In prespecified mediation analyses, we fit latent growth structural equation mediation models to test our theory of change that intervention related increases in well-being skills mediate 37-week follow-up improvements in distress and well-being.^18^ Mediators were modeled as a latent intercept and slope, controlling for baseline outcome score. Mediator residual variances were constrained to be time invariant. Model fit was assessed using standard relative and absolute fit indices.^35,36^ Asymmetric, bias-corrected bootstrapped (5000) confidence intervals were used.^37^ In exploratory analyses, we estimated intervention effects on anxiety and depression, and tested whether administrators moderated treatment effects.

Deviating from our prespecified plan, we did not collect race and ethnicity or socially desirable responding because of poor scale psychometrics in pilot data. The former was not collected because it is uncommonly assessed in Mexico and partners raised concerns. Additionally, because postintervention to follow-up change was approximately linear, we did not as planned estimate interaction effects at the 25-week intermediate follow-up assessment for primary and burnout outcomes (eFigures 1 and 2 in Supplement 2).

## Results

**Figure 1.**
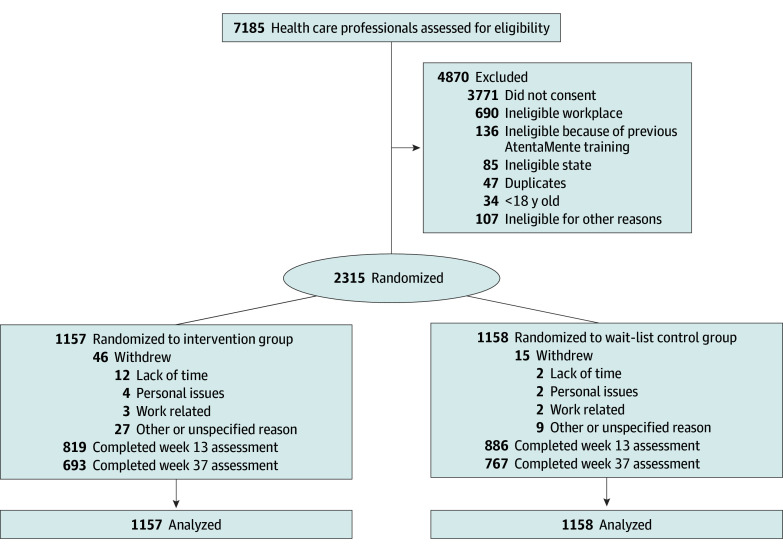
Flow of Participants Through the Study

In this randomized clinical trial, 7185 participants completed the prescreen between March 15 and May 21, 2023; of these, 6021 were deemed preliminarily eligible, 2989 consented to participate, and 2315 completed the baseline survey between April 24 and May 21, 2023. The participation rate among preliminarily eligible participants was 38.5% (2315 of 6201). On May 23, 2023, 2315 participants were randomly assigned (n = 1157 intervention, n = 1158 control) (Figure 1). Participants worked in 729 hospitals or clinics and 33 regional or state administrative offices, representative of the diversity of Mexican health care contexts. Among the 2315 participants, 1693 (73.1%) identified as female and 614 (26.5%) as male; 778 (33.4%) were nurses, 736 (31.7%) were physicians, 187 (8.1%) were psychologists, 183 (7.9%) were administrators, 154 (6.7%) were dentists, 75 (3.2%) were social workers, 64 (2.8%) were nutritionists, 55 (2.4%) were laboratory technicians, 40 (1.7%) were physical therapists, 29 (1.3%) were health promoters, and 8 (<0.1%) were other health professionals^38^ (Table 1). The mean (SD) age was 33.7 (11.4) years. At postintervention end point, 73.7% of participants provided data (1705 of 2315; 70.8% intervention [819 of 1157], 76.5% control [886 of 1158]); and at the follow-up end point, 63.1% of participants provided data (1460 of 2315; 59.9% intervention [693 of 1157], 66.2% control [886 of 1158]).

**Table 1.** Characteristics of Mexican Health Care Professional Participants

Characteristic	Health care professionals, No. (%)	
	Intervention (n = 1157)	Wait-list control (n = 1158)
Sex		
Female	845 (73)	848 (73)
Male	308 (27)	306 (26)
Preferred not to answer	4 (<1)	4 (<1)
Age, y		
18-19	10 (1)	10 (1)
20-29	522 (45)	511 (44)
30-39	273 (24)	255 (22)
40-49	203 (18)	223 (19)
50-59	112 (10)	125 (11)
60-69	35 (3)	33 (3)
70-79	2 (<1)	1 (<1)
Socioeconomic level^a^		
A/B	574 (50)	551 (48)
C+	292 (25)	321 (28)
C	147 (13)	138 (12)
C-	98 (8)	94 (8)
D+	34 (3)	35 (3)
D	12 (1)	18 (2)
E	0 (<1)	1 (<1)
Profession		
Nurse	402 (35)	376 (32)
Physician	377 (33)	359 (31)
Psychologist	90 (8)	97 (8)
Administrator	96 (8)	87 (8)
Dentist	64 (6)	90 (8)
Social work	38 (3)	37 (3)
Nutritionist	33 (3)	31 (3)
Laboratory technician	25 (2)	30 (2)
Physical therapist	15 (1)	25 (3)
Health promotor	14 (1)	15 (1)
Other	3 (<1)	5 (<1)

^a^ Socioeconomic status is coded according to the Asociación Mexicana de Agencias de Inteligencia de Mercado y Opinión index^38^ (eTable 8 in Supplement 2). The other profession category primarily consisted of positions that did not fit in another category.

Most intervention participants attended or viewed at least 7 of 9 sessions (733 of 1157 [63.4%]), but only 331 of 1157 (28.6%) used the app for 300 minutes or longer, with 334 of 1157 (29.1%) logging 0 minutes. Consequently, 280 of 1157 intervention participants (24.2%) adhered to communicated engagement standards. Across the 9 sessions, between 58.4% to 65.3% of participants engaged asynchronously by watching recordings of the sessions. Intercohort fidelity of intervention content implementation was 95% to 99%, according to auditors.

**Figure 2.**
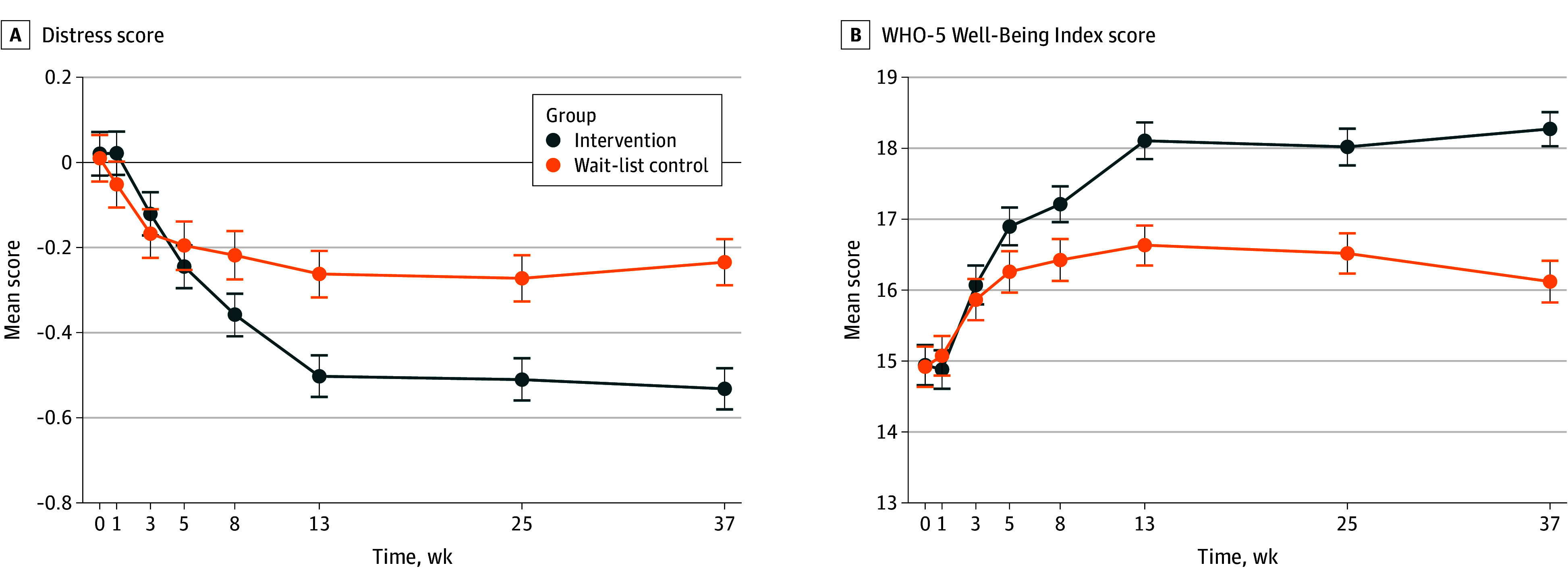
Change in Primary Outcomes by Group Week 0 is baseline, prior to random assignment. Weeks 1, 3, 5, 8, 13, 25, and 37 are the postrandomization assessments. Week 13 postintervention and week 37 follow-up are the prespecified primary end points. Error bars indicate 95 CIs. WHO-5 indicates World Health Organization–5.

Outcome descriptive statistics by group at study end points are reported in Table 2 (eTable 2 in Supplement 2 for all time points). In primary analyses, the intervention was superior to control in reducing distress at postintervention (adjusted SMD, −0.24; 95% CI, −0.32 to −0.16; FDR-corrected *P* < .001; and at 37-week follow-up adjusted SMD, −0.36; 95% CI, −0.44 to −0.28; FDR-corrected *P* < .001). The intervention also increased well-being at postintervention (adjusted SMD, 0.27; 95% CI, 0.18 to 0.35; FDR-corrected *P* < .001) and at 37-week follow-up (adjusted SMD, 0.42; 95% CI, 0.34 to 0.50; FDR-corrected *P* < .001) (Figure 2).

**Table 2.** Study Outcomes at 13 Weeks’ and 37 Weeks’ Postrandomization for Intervention and Control Groups

Week	Intervention		Wait-list control		Mean difference (95% CI)	Adjusted mean difference (95% CI)	Effect size (95% CI)^a^		*P* value
	No./No. (%)	Mean (SD)	No./No. (%)	Mean (SD)					
**Distress: *z*-scored and aggregated Perceived Stress scale, PROMIS Anxiety, and PROMIS Depression scales**									
Baseline	1157/1157 (100.0)	0.01 (0.89)	1158/1158 (100.0)	0 (0.94)	0.01 (−0.06 to 0.08)	NA	NA		NA
Week 13	819/1157 (70.8)	−0.52 (0.84)	886/1158 (76.5)	−0.28 (0.95)	−0.24 (−0.33 to −0.15)	−0.22 (−0.28 to −0.15)	−0.24 (−0.31 to −0.17)		<.001
Week 37	693/1157 (59.9)	−0.55 (0.84)	767/1158 (66.2)	−0.25 (0.94)	−0.30 (−0.39 to −0.21)	−0.33 (−0.41 to −0.24)	−0.36 (−0.44 to −0.27)		<.001
**WHO-5 Well-being (possible range, 5 to 25)**									
Baseline	1157/1157 (100.0)	14.86 (4.89)	1158/1158 (100.0)	14.84 (4.98)	0.02 (−0.38 to 0.42)	NA	NA		NA
Week 13	821/1157 (71.0)	18.02 (4.46)	887/1158 (76.6)	16.54 (4.92)	1.47 (1.02 to 1.92)	1.30 (0.93 to 1.68)	0.27 (0.19 to 0.34)		<.001
Week 37	697/1157 (60.2)	18.18 (4.14)	768/1158 (66.3)	16.03 (5.08)	2.15 (1.68 to 2.62)	2.07 (1.97 to 2.18)	0.42 (0.34 to 0.50)		<.0001
**Healthy Minds Index awareness (possible range, 1 to 5)**									
Baseline	1157/1157 (100.0)	2.46 (0.73)	1158/1158 (100.0)	2.5 (0.78)	−0.05 (−0.11 to 0.01)	NA	NA		NA
Week 13	817/1157 (70.6)	2.77 (0.72)	881/1158 (76.1)	2.65 (0.77)	0.12 (0.05 to 0.19)	0.15 (0.10 to 0.21)	0.20 (0.13 to 0.28)		<.001
**Healthy Minds Index connection (possible range 1 to 5)**									
Baseline	1157/1157 (100.0)	2.59 (0.66)	1158/1158 (100.0)	2.57 (0.7)	0.02 (−0.04 to 0.08)	NA	NA		NA
Week 13	817/1157 (70.6)	2.75 (0.71)	881/1158 (76)	2.63 (0.75)	0.12 (0.05 to 0.19)	0.09 (0.04 to 0.15)	0.13 (0.05 to 0.22)		.001
**Healthy Minds Index insight (possible range 1 to 5)**									
Baseline	1157/1157 (100.0)	2.41 (0.83)	1158/1158 (100.0)	2.41 (0.85)	0 (−0.07 to 0.07)	NA	NA		NA
Week 13	817/1157 (71)	2.74 (0.76)	881/1158 (76.1)	2.57 (0.83)	0.17 (0.09 to 0.25)	0.19 (0.12 to 0.26)	0.23 (0.14 to 0.31)		<.001
**Healthy Minds Index purpose (possible range 1 to 5)**									
Baseline	1157/1157 (100.0)	3.29 (0.79)	1158/1158 (100.0)	3.29 (0.82)	0 (−0.07 to 0.07)	NA	NA		NA
Week 13	817/1157 (70.6)	3.47 (0.74)	881/1158 (76.1)	3.35 (0.82)	0.11 (0.04 to 0.18)	0.13 (0.07 to 0.19)	0.16 (0.09 to 0.23)		<.001
**GQ-6 Gratitude (possible range 1 to 7)**									
Baseline	1157/1157 (100.0)	5.89 (0.88)	1158/1158 (100.0)	5.85 (0.94)	0.04 (−0.03 to 0.11)	NA	NA		NA
Week 13	817/1157 (70.6)	5.99 (0.91)	875/1158 (75.6)	5.91 (0.93)	0.08 (−0.01 to 0.17)	0.01 (−0.04 to 0.02)	0.01 (−0.04 to 0.02)		.53
**Maslach Burnout Inventory emotional exhaustion (possible range, 0 to 54)**									
Baseline	1157/1157 (100.0)	24.53 (12.77)	1158/1158 (100.0)	24.27 (12.93)	0.26 (−0.79 to 1.31)	NA	NA		NA
Week 13	814/1157 (70.4)	21.09 (12.39)	872/1158 (75.3)	22.88 (12.89)	−1.79 (−3 to −0.58)	−1.59 (−2.52 to −0.66)	−0.12 (−0.20 to −0.05)		<.001
Week 37	688/1157 (59.5)	18.25 (12.18)	767/1158 (66.2)	21.11 (13.07)	−2.86 (−4.16 to −1.56)	−2.45 (−2.56 to −2.34)	−0.19 (−0.27 to −0.11)		<.001
**Maslach Burnout Inventory personal accomplishment (possible range, 0 to 48)**									
Baseline	1157/1157 (100.0)	40.3 (6.93)	1158/1158 (100.0)	40.53 (6.93)	−0.23 (−0.79 to 0.33)	NA		NA	NA
Week 13	814/1157 (70.4)	40.98 (7.29)	872/1158 (75.3)	40.69 (7.23)	0.29 (−0.4 to 0.98)	0.47 (−0.15 to 1.09)		0.07 (−0.02 to 0.16)	.15
Week 37	688/1157 (59.5)	40.95 (7.85)	767/1158 (66.2)	39.87 (8.42)	1.08 (0.24 to 1.92)	1.22 (1.13 to 1.31)		0.18 (0.09 to 0.26)	<.001

Abbreviations: GQ-6, Gratitude Questionnaire–6; NA, not applicable; PROMIS, Perceived Stress and Patient Reported Outcomes Measurement Information System; WHO-5, World Health Organization–5.

Intervention is the Integrated Stress Toolbox for Healthcare Providers treatment group. Baseline data was collected prior to random assignment. Week 13 is postintervention. Week 37 is 6-month follow-up. See eTable 8 in Supplement 2 for descriptive data on all time points. All *P* values are false discovery rate–corrected.

^a^ Cohen *d*.

In exploratory analyses disaggregating distress into anxiety and depressive symptoms, intervention participants reported significant postintervention reductions in anxiety (adjusted SMD, −0.24; 95% CI, −0.32 to −0.16; *P* < .001) and depressive symptoms (adjusted SMD, −0.22; 95% CI, −0.30 to −0.14; *P* < .001), with larger magnitude reductions at follow-up for both anxiety (adjusted SMD, −0.31; 95% CI, −0.39 to −0.23; *P* < .001) and depressive symptoms (adjusted SMD, −0.33; 95% CI, −0.41 to −0.24; *P* < .001). Intervention but not control group reductions in anxiety and depressive symptoms at both endpoints exceeded MCID thresholds (eTable 3 in Supplement 2).^39,40^

**Figure 3.**
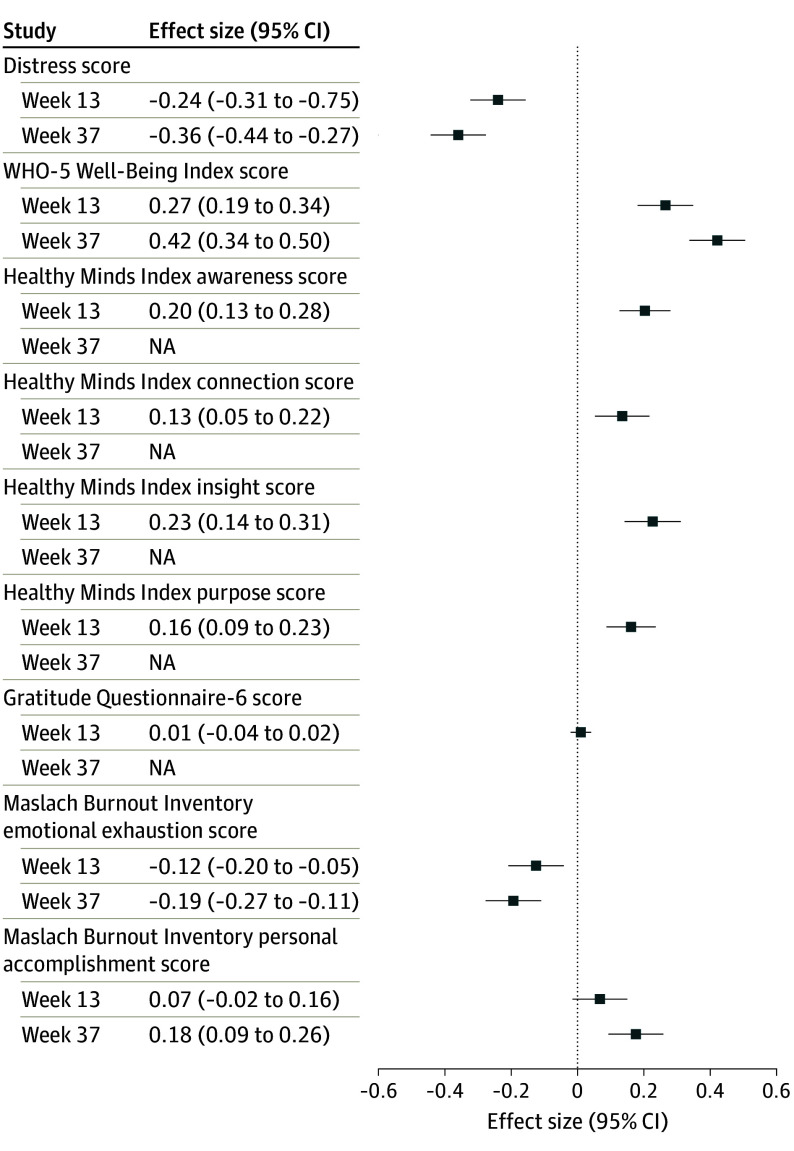
Effect Sizes for All Outcomes at Study End Points All effect sizes favored the Integrated Stress Toolbox for Healthcare Providers treatment group (intervention). For distress and emotional exhaustion burnout, intervention relative to control reductions were considered favorable. For all other measures, intervention relative to control increases were considered favorable. Emotional exhaustion and personal accomplishment occupational burnout were the only secondary outcomes assessed at follow-up. NA indicates not applicable; WHO-5, World Health Organization–5.

The intervention was superior to control in significantly improving all secondary outcomes at postintervention (adjusted SMDs ranged from 0.13 to 0.23; all FDR-corrected *P* ≤ .001) except personal accomplishment burnout and gratitude. At follow-up, the intervention was superior to control in improving both assessed secondary outcomes: emotional exhaustion burnout (adjusted SMD, −0.19; 95% CI, −0.27 to −0.11; FDR-corrected *P* < .001) and personal accomplishment at work (adjusted SMD, 0.18; 95% CI, 0.10 to 0.26; FDR-corrected *P* < .001) (Table 2, Figure 3).

### Adverse Events

There were 10 adverse events (AEs; 10 of 2315 [0.04%]; 6 in intervention, 4 in control) (eTable 4 in Supplement 2). Of the 6 AEs reported among 1157 intervention participants (0.05%), one was serious (ie, death due to hypertensive related cerebral hemorrhage) and deemed unrelated to the intervention. Two of the 6 intervention group AEs and 1 of the 4 control group AEs were deemed study related but not serious.

In prespecified exploratory moderation analyses, we observed no evidence of treatment effect moderation by participant age, gender, location (ie, state), or profession type. In prespecified sensitivity analyses, intervention effects on primary outcomes were robust to all tested missing data assumptions (eTable 5 in Supplement 2). All intervention effects at postintervention on secondary outcomes were robust to sensitivity analyses except for connection (10% and 20% worse than imputed) and emotional exhaustion (20% worse than imputed). At follow-up, intervention effects on emotional exhaustion but not personal accomplishment were robust to sensitivity analyses. In an exploratory analysis, HCP role (provider vs administrator) did not significantly moderate the effect of the intervention, though in stratified analyses, clinicians’ outcomes were qualitatively better than those of administrators (eTable 6 in Supplement 2).

Prespecified mediation models controlling for baseline score on the outcome fit the data well (eTable 7 in Supplement 2). Increased attention and awareness of thoughts over the intervention period mediated 72.3% of the intervention’s effect at follow-up on distress (*P* < .001) and 42.2% of the intervention’s effect on well-being (*P* < .001). Increased care for others (ie, connection skill) over the intervention period mediated 22.9% of the intervention’s effect at follow-up on distress (*P* = .005) and 12.0% of the intervention’s effect on well-being (*P* = .009). Increased self-reflection (ie, insight skill) over the intervention period mediated 49.0% (*P* < .001) of the intervention’s effect at follow-up on distress and 30.7% (*P* < .001) of the intervention’s effect on well-being (*P* < .001). Increased meaning and purpose in daily activities over the intervention period mediated 36.4% of the intervention’s effect at follow-up on distress (*P* < .001) and 24.4% of the intervention’s effect on well-being (*P* < .001).

## Discussion

A digital well-being training implemented at a large scale significantly reduced Mexican HCP distress and increased well-being, with improvements increasing in magnitude over the 37-week follow-up. Disaggregating the distress outcome, intervention group improvements in anxiety and depressive symptoms exceeded MCID thresholds whereas control group improvements did not. As hypothesized, significant intervention group strengthening of well-being skills over the intervention period mediated reduced distress and increased well-being at 37-week follow-up. Increased awareness mediated a particularly large proportion of the direct effect of intervention assignment on distress (72.3%). In addition, at follow-up the intervention group improved on the 2 assessed facets of occupational burnout: emotional exhaustion and personal accomplishment. Most intervention-related improvements were robust to sensitivity analyses. These results suggest that it is possible to modestly but meaningfully improve the well-being of Mexican HCPs through digital intervention at scale, perhaps by strengthening well-being skills, with benefits that appear consistent across HCPs (eg, gender).

Although the intervention group reported significant improvements on most outcomes, the magnitude of improvements was small to small-to-moderate by conventional benchmarks.^41^ Coupled with the wait-list control design, caution is warranted, as research has demonstrated that this design often results in larger magnitude effects than active or placebo-controlled trials.^42^ On the other hand, the observed effects should be interpreted in light of evidence that intervention effects decrease, sometimes substantially, across a range of outcomes as sample size increases, and even small magnitude positive changes at the population level can be meaningful.^43,44,45^

Developing and testing well-being programs that HCPs will use is challenging. Here, only 24.2% of intervention participants adhered to requested engagement levels. Technical issues downloading/activating the app were major barriers. Apps are often presented as scalable interventions. Although technical support was provided, in certain contexts or populations, app-based interventions may be less feasible than expected and other forms of digital intervention may be more accessible. Across the 9 intervention sessions, 58.4% to 65.3% of participants engaged asynchronously by watching recordings of sessions. The high level of asynchronous engagement indicates that flexible options for participation may be important for Mexican HCPs with demanding schedules.

The intervention was superior to control in improving multiple dimensions of well-being despite many participants not reaching requested engagement levels, indicating strong potential for optimization, perhaps by reducing dosage or complexity. Finding the optimal balance between program intensity and effectiveness is essential; time constraints are a source of HCP stress and will likely continue to be a barrier to engaging with well-being training. In this regard, partnering health system leaders were forward thinking. While the intervention was provided to individuals, it was sponsored as professional development, illustrating the benefits of organizational support for HCP well-being.

### Limitations

Study results are limited by the reliance on participant-reported outcomes, the lack of condition masking and the non-active/placebo-controlled design. The major concerns presented by these limitations are the possibility of response bias and placebo effects. To ensure intervention effects were not driven by response bias or placebo, future active/placebo-controlled trials that include nonparticipant-reported outcomes are needed. Additional limitations included (1) assessing 2 of 3 burnout facets; (2) the inability to draw causal mechanistic inferences from mediation models; (3) despite high levels of asynchronous engagement, the possibility that instructor training requirements limit future scaling up; and (4) questions regarding study generalizability. Although the enrolled sample was large and the study highly powered, because enrollment was open to 4000 HCPs but 2315 enrolled, it is possible that enrolled HCPs were not representative of the HCP population.

## Conclusions

In this large RCT of a digital well-being training, we observed promising evidence that HCP well-being can be modestly but meaningfully improved, potentially through the strengthening of well-being skills, with little evidence for safety concerns. This study illustrates a potentially useful model of health care systems support for HCPs.

## Notes

Supplement 1.
Trial Protocol

Supplement 2. **eTable 1.** Table of assessments **eMethod 1.** R statistical software packages used in the analyses reported in this research **eMethod 2.** Careless responding analyses **eFigure 1.** Trajectories of change on wellbeing skills by group, using observed data **eFigure 2.** Trajectories of change on burnout and gratitude by group, using observed data **eTable 2.** Primary and secondary outcomes at all timepoints by group **eTable 3.** Change in anxiety and depression symptoms by group **eTable 4.** Adverse events table **eTable 5.** Results of sensitivity analyses **eTable 6.** Descriptives statistics by group and status as an administrator **eTable 7.** Fit statistics from latent growth structural equation mediation models **eTable 8.** Description of socioeconomic levels coding

Supplement 3.
Nonauthor Collaborators

Supplement 4.
Data Sharing Statement
