# Supplementary material for: Digital Well-Being Training With Health Care Professionals: A Randomized Clinical Trial
Source: JAMA Intern Med. 2025 Aug 18;185(10):1248–56. doi: 10.1001/jamainternmed.2025.3888 (PMC12362274; doi:10.1001/jamainternmed.2025.3888)
Supplement: Supplement 1. — Trial Protocol [file jamainternmed-e253888-s001.pdf]

PROTOCOL TITLE:
Stress Toolbox for Mexican Healthcare Providers

PRINCIPAL INVESTIGATORS:
Richard Davidson, PhD
Center for Healthy Minds, UW-Madison
(608) 262-8189
rjdavids@wisc.edu

Leandro Chernicoff, PhD
Atentamente Consultores A.C.,
(608) 265-3610
chernicoff@wisc.edu

LEAD RESEARCHER:
Matthew Hirshberg, PhD
Center for Healthy Minds, UW-Madison
(608) 262-0035
hirshberg@wisc.edu

GRANT TITLE:
Training character in Mexican healthcare providers as a pathway to mental health and well-being

GRANT NUMBER:
TWCF0630

FUNDING SOURCE:
Templeton World Charity Foundation

IRB STUDY ID:
2023-0990

CLINICALTRIAL.GOV ID:
NCT05767970

VERSION NUMBER/DATE:
1.0; 01.10.2023
1.1; 12.20.2024 Update to actual study dates. Correct statistical plan consistent with actual number of statistical tests (See also https://osf.io/9g58j).

CONTENTS

1. INTRODUCTION
2. STUDY OBJECTIVES
3. BACKGROUND
4. STUDY DSIGN
4.1 NUMBER OF SUBJECTS
4.2 SUBJECT SELECTION
4.2.1 Inclusion and Exclusion Criteria
4.3 SCREENING, RECRUITMENT, AND ENROLLMENT
4.3.1 Screening
4.3.2 Recruitment and Enrollment
4.3.3 Rescreening
4.3.4 Early Withdrawal of Participants
5. STUDY INTERVENTION
6. OUTCOME MEASURES
6.1 DEFINITION AND ASCERTAINMENT OF OUTCOMES
6.1.1 Primary Outcomes
6.1.2 Secondary Outcomes
6.1.3 Tertiary (Mechanistic) Outcomes
7. STUDY PROCEDURES AND VISITS
7.1 RANDOMIZATION
7.2 SCHEDULE OF EVENTS
8. STATISTICAL ANALYSIS, SAMPLE SIZE AND POWER CALCULATIONS
9. DATA MANAGEMENT
9.1 DATA MONITORING PROCEDURES
10. SAFETY AND ADVERSE EVENTS
10.1 DEFINITIONS
11. ETHICAL AND REGULATORY CONSIDERATIONS
11.1 INFORMED CONSENT
11.2 INSTITUTIONAL REVIEW BOARD
11.3 RISK TO HUMAN SUBJECTS
11.3.1 Protections against risk
11.3.2 Potential benefits of the proposed research to human participants and others
11.3.3 Importance of the knowledge to be gained
12. STUDY DOCUMENTS
12.1 RETENTION OF RECORDS
13. PUBLICATION AND DISSEMINATION POLICY

1. INTRODUCTION

This document is a protocol for human subjects research to be conducted in accordance with US and international law, and the WMA Declaration of Helsinki.

2. STUDY OBJECTIVES

The proposed parallel-group randomized clinical trial will test the efficacy of the Integrated Toolbox for Healthcare Providers (ISTH) on the psychological functioning, well-being, occupational performance, and peripheral inflammation of Mexican healthcare providers.

3. BACKGROUND

The COVID-19 pandemic has placed an extraordinary strain on health care systems and providers. According to Amnesty International (Amnesty International, 2021), as of figures compiled by March 5th, 2021, at least 3,371 Mexican healthcare providers (HCPs) have died from COVID-19. These numbers rank Mexico second in the world in terms of total HCP deaths, second only to the United States with 3,507 reported deaths, but with 2.5 times the deaths per capita (2.6 to 1.07 HCP deaths per 100,000 population, respectively). According to the John Hopkins coronavirus resource center, Mexico also has the highest case-fatality ratio in the world with 9 deaths per 100 confirmed cases as of March 27th (John Hopkins Coronavirus Resource Center, 2021). Though these numbers should be interpreted with caution, they point to the alarming effects that the COVID-19 pandemic is having on Mexican HCPs and its healthcare system.

Strategies to mitigate the impacts of COVID-19 on HCP mental health in Mexico have been limited. The COVID-19 pandemic has catalyzed a mental health crisis in the Mexican healthcare system. Hoping to mitigate this issue, Mexican health authorities established call centers offering psychological support for HCPs. Unfortunately, the strategy has had limited impact as evidenced by the low number of calls received in the first six months of the program (less than 200 in the state of Jalisco which has over 25,000 health care workers). Research suggests that HCPs are particularly likely to be deterred from seeking help for mental health problems due to concerns of disclosure/confidentiality, negative social judgement, and stigma (Clement et al., 2015). This resistance to seeking help might be amplified by a culture of ‘invincibility’ within medicine (Henderson et al., 2012). Thus, a less stigmatizing form of care, one that doesn’t talk about mental illness but rather about character strengths, well-being, and how to better cope with stress and burnout, might be a more suitable form of intervention to help HCPs.

The Stress Toolbox for Healthcare Providers (STH).

To address HCP mental health and rooted in the belief that character development is vital to coping with stressful events, to resilience (Harzer & Ruch, 2015), to professional fulfillment (Brown & Gunderman, 2006; Hausler et al., 2017; Kachel, Huber, Strecker, Höge, & Höfer, 2020), and to human flourishing (Peterson & Seligman, 2004; Seligman, 2018), AtentaMente developed the “Stress Toolbox for Health Providers” (STH). The STH is a Zoom-based, 16-hour course (eight x 2-hour weekly sessions) aimed at strengthening four pillars of character that underlie well-being: Awareness, Connection, Insight, and Purpose (ACIP) competencies. Recent evidence suggests that these are key pillars of well-being and critically, that they demonstrate plasticity and therefore the opportunity for intentional training and development (Dahl, Wilson-Mendenhall, & Davidson, 2020). Awareness competencies include attention regulation, mindfulness, and meta-awareness; Connection competencies include empathy, kindness, and gratitude; Insight competencies include self-knowledge and self-inquiry, a curiosity-driven

investigation of self-related beliefs and processes; and Purpose competencies include knowing what brings meaning, purpose, and fulfillment in one's life.

The STH draws from gold-standard, evidence-based mental health and stress coping interventions, such as Mindfulness-based Stress Reduction (MBSR; Kabat-Zinn, 1983), character interventions (Niemiec, 2017), cognitive-behavior therapy (Beck, 2011), rational emotive behavior therapy (Ellis & MacLaren, 1998), and acceptance and commitment therapy (Bond, Hayes, Lillis, Luoma, & Masuda, 2006). For example, the STH includes mindfulness of breathing and body scan from MBSR; identification and questioning of cognitive distortions, and positive reappraisal from CBT and REBT; values clarification and committed action from ACT; and gratitude and mindful listening and speaking from CI. Developed by native Spanish speakers in Spanish for a Latin American audience, the STH has been culturally adapted for its intended audience (Benish, Quintana, & Wampold, 2011). Cultural adaptation of behavioral interventions has been shown to improve efficacy (Benish et al., 2011). The result is a training that is culturally relevant, comprehensive, simple, and easy to learn and apply.

To date, the STH has been implemented with over 3200 Mexican HCPs (i.e., physicians, nurses, residents, social workers, psychologists, lab technician, technicians, and administrative staff). In a pilot study with 502 HCPs conducted between October and December 2020, feasibility and acceptability were evidenced by the 81% of participants who completed at least 6 of 8 sessions, and the high ratings of program content, instruction, and perceived benefits (9.8/10). Preliminary data on program efficacy collected from 258 participants who volunteered to provide pre- and post-program data are encouraging. Although effects sizes are likely inflated through selection bias, participants reported significant pre- to post-test reductions in depression (d=0.81), anxiety (d=0.58), stress (d=0.65), and occupational burnout (emotional exhaustion d=1.20; personal accomplishment d=0.98; Supplementary Materials).

Healthy Minds Program

The Center for Healthy Minds (CHM) at the University of Wisconsin-Madison has recently developed a character-based mHealth meditation intervention called the Healthy Minds Program (HMP, Figure 2), organized around the ACIP model of well-being (Dahl et al., 2020). The HMP app is freely available throughout the world. A unique feature of the HMP is that it has been designed to be used amidst the business of daily life. Short, podcast style lessons on the science of well-being from world-renowned scientists are paired with instruction in meditation practices that are highly flexible. Guided practice can be done as a sitting meditation or active practice, with the latter designed to be carried out during simple daily activities, such as eating or going for a walk. Individuals can also choose between different guides and different lengths, ranging from 5 to 30 minutes, allowing maximum individualization.

Two randomized controlled trials (RCTs) of the HMP have demonstrated that even at very low dosages (on average <5 min/day for one month), the program results in a range of positive outcomes related to mental health, human flourishing, and character strengths. Goldberg and colleagues (Goldberg et al., 2020) examined an 8-week iteration of the HMP in the general population (N=343) and reported significant HMP effects on psychological distress and loneliness. Hirshberg et al. (M. J. et al. Hirshberg, 2021) conducted a fully remote RCT with public school educators (N=662) during the COVID-19 pandemic. Using a 4-week version of the HMP, they reported significant HMP effects on the primary outcome of psychological distress and on all secondary ACIP character outcomes (e.g., mindfulness, self-compassion, cognitive distancing). All intervention effects persisted at the three-month follow-up compared to the wait list control group. Also important, no evidence for adverse effects were found, and the benefits

of the app were at least as large in the subsample of participants reporting elevated anxiety and depression at baseline (>50% of the sample).

Integrating the STH and the HMP.

The Center for Healthy Minds (CHM) and AtentaMente will integrate the STH with the HMP. The resulting ISTH program will be a 12-week intervention that consists of an initial eight-week synchronous and app-supported training, followed by an additional four-weeks of app-only training. Employing the best practices both CHM and AtentaMente have honed over past projects, the synchronous components will be delivered through Zoom and recorded, to allow for asynchronous participant engagement if required, and app implementation will be carried out in accordance with the strategies Hirshberg et al. (2021) used successfully (most important is immediate contact with non-adhering participants). The ISTH has the potential to represent a significant step toward fulfilling the urgent need to support HCP well-being in Mexico and longer-term, Latin America.

The STH and the HMP are well-suited to integration. They are constructed around the same theory of change (Figure 1) and model of well-being (i.e., ACIP model), make use of declarative and procedural learning strategies, and provide instruction in many of the same foundational meditation practices. For example, both interventions employ short lessons that teach the key principles underlying a character strength leading to declarative learning about this strength, and follow declarative learning with guided meditations and exercises tailored to daily life experiences where the strength is practiced and applied (procedural learning). The major work of this integration is synchronizing the scope and sequence of the two programs so that users receive a seamless, uniform program, and constructing a modest amount of new content (4 new “learn” podcasts, eight new guided practices) for the four-week fully remote component of the training.

Figure 1. Theory of Change

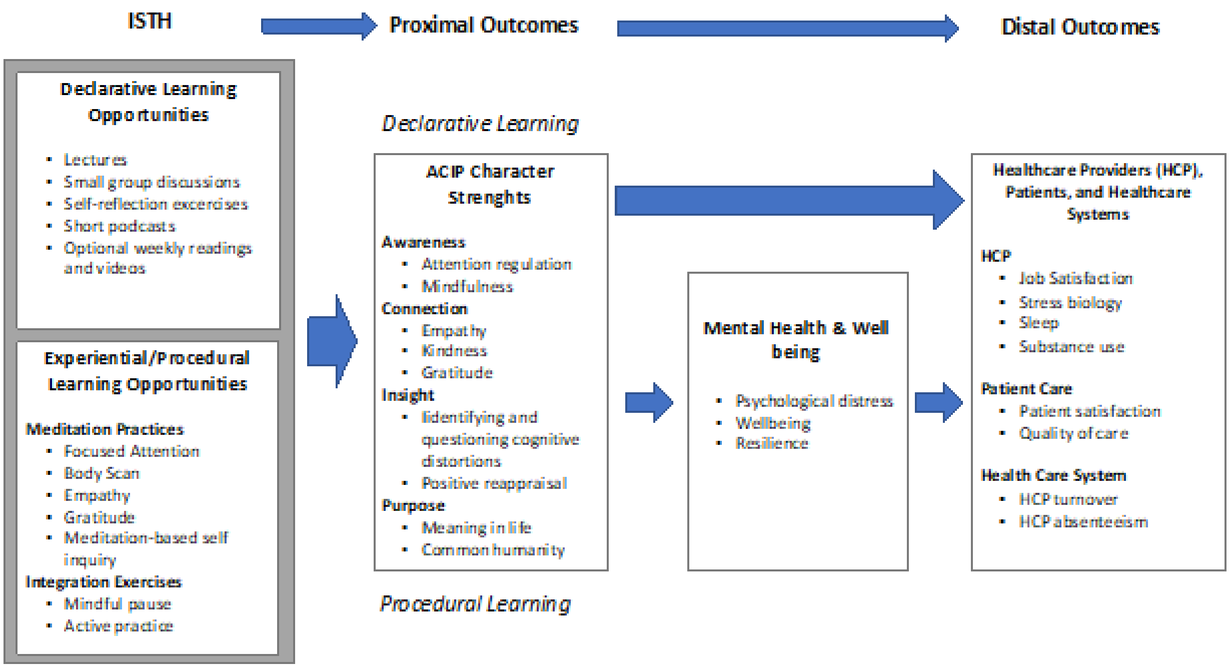

4. STUDY DESIGN

A two-arm parallel RCT with a wait-list controlled design in which the control group may pursue professional development activities as usual and will receive access to the intervention following the final study assessment nine-months post-randomization. Assessors will be blind to group assignment. Target sample sizes were determined based on power analyses. The unit of randomization is the individual participant. The co-primary outcomes are psychological distress and well-being. Participants will complete assessments at baseline, prior to random assignment, after weeks one, three, five, eight and twelve (i.e., post-intervention), and after weeks 24 and 36. A subset of 510 participants (255 from each condition) will be selected for a dried blood spot substudy in which dried blood samples will be collected at baseline, week 12 and week 24. A subset of 510 participants, ideally overlapping with the dried bloodspot substudy, will be selected for the patient satisfaction substudy in which enumerators assess participants' patients perspectives on their care.

4.1 Number of subjects

Data will be collected from an anticipated sample of up to 4,000 Mexican healthcare providers who work in a participating healthcare system within a participating Mexican State (Campeche, Coahuila, Guanajuato, Jalisco, Nuevo Leon, Oaxaca, Queretaro, or Sonora).

4.2 Subject Selection and Withdrawal

4.2.1.1 Inclusion and Exclusion Criteria

Inclusion Criteria:

- $\geq$ 18 years old
- Employee in a participating healthcare system in a participating Mexican State

Exclusion Criteria:

- <18 years old
- Not in a participant healthcare system in a participating state
- Does not work in a qualifying healthcare provider role
- Does not have regular access to reliable internet and/or a smartphone capable of downloading the Healthy Minds Program app

4.3. Screening, Recruitment, and Enrollment

4.3.1 Screening

Participants will enter into the study REDCap project and complete a preliminary eligibility screen. Those deemed eligible will be sent a follow-up invitation to provide additional information to determine final study eligibility and be provided a detailed summary of study activities and the study timeline.

4.3.2 Recruitment and Enrollment

Recruitment will occur through informational sessions at large regional healthcare facilities and internal communications from the Minister's of Health in each participating state. Participants will self-enroll via the REDCap study project as described in screening.

4.3.4 Early Withdrawal

Participants may withdraw at any time by requesting withdrawal from the study team.

5 STUDY INTERVENTION

The Integrated Stress Toolbox for Healthcare providers (ISTH) is a 12-week meditation-based well-being training. In weeks 1-8, participants engage in weekly 2-hour class sessions that are recorded and posted on a private Youtube channel. There is a final two-hour session at week 12. In-class learning is augmented and extended through content in a special version of the Healthy

Minds Program smartphone app that participants are asked to use on a daily basis throughout the intervention.

6 OUTCOME MEASURES

6.1 DEFINITION AND ASCERTAINMENT OF OUTCOMES

Outcomes were selected to assess the general and occupational specific psychological factors theoretically and empirically associated with improved mental health, well-being and occupational outcomes.

6.1.1 Primary Outcomes

Study co-primary outcomes are distress, operationalized as the z-scored aggregate of Patient-Reported Outcomes Measurement Information System (PROMIS) Depression (8-items) and Anxiety (8-items) Scales, and the NIH Toolbox Perceived Stress v2.0 (10-items) and well-being, measured with the World Health Organization Five Well-Being Index (WHO-5). The WHP-5 is a short self-reported measure of current mental well-being (time frame the previous two weeks). Higher distress scores indicate greater symptoms of psychological distress and higher well-being scores (range 0 lowest possible well-being to 25 highest possible well-being) indicate greater well-being.

6.1.2 Secondary Outcomes

Secondary Outcomes consist of: the Healthy Minds Index (17-items), a measure of four components of well-being – awareness, connection insight and purpose; Gratitude Questionnaire 6 (6-items); and the Maslach Burnout Inventory Emotional Exhaustion and Personal Accomplishment subscales (15-items).

Administrative records. We will receive prior, current and next year employment records on participants, including, number and reasons for absences and type of employment. Biomarkers - Dried blood spot substudy: CRP, IL-6, IL-10, TNFalpha.

6.1.3 Tertiary (Mechanistic) Outcomes

Five Facet Mindfulness Questionnaire Act with Awareness and Nonreact to inner experience subscales (FFMQ; Baseline, after weeks 3, 8, and 12); Interpersonal Reactivity Index Empathic Concern subscale (IRI-Empathic Concern; baseline, after weeks 5, 8 and 12); Cognitive Emotion Regulation Questionnaire reappriasal subscale (CERQ- Emotion regulation; baseline, after weeks 3, 5, 8, and 12); Meaning in Life Questionnaire Presence subscale (ML– Presence; baseline, after weeks 1, 8 and 12); MAIA self-regulation subscale (baseline, after weeks 1, 3, 8, 12); Compassionate Engagement and Action Scale Compassion for others subscale (CEAS; baseline, after weeks 8 and 12); Global Assessments of Character Strengths 24 (GACS; baseline, after weeks 12 and 36).

7 STUDY PROCEDURES AND VISITS

7.1 Randomization

Using stratified random permuted blocks of 4, with Mexican State and the tier of healthcare facility as the strata, participants will be randomly assigned 1:1 to either the ISTH or wait-list control. The randomization list will be entered into REDCap and participants will be auto-assigned based on strata via REDCap’s assignment features (i.e., no experimenter involvement).

7.2 Schedule of Events

Event	Prescreen	Baseline	Wk 1	Wk 3	Wk 5	Wk 8	Wk 12	Wk 24	Wk 36
Screening and Stratification Measures									
Informed Consent		X							
Demographics	X	X							
Primary and Secondary Outcomes									
PROMIS Anxiety, Depression & Perceived Stress		X	X	X	X	X	X	X	X
WHO-5		X	X	X	X	X	X	X	X
Healthy Minds Index		X	X		X	X	X		
GQ-6		X			X	X	X		
Maslach Burnout Inventory		X				X	X	X	X
Dried blood spots		X					X	X	
Tertiary Outcomes (Mechanisms)									
FFMQ		X		X		X	X		
IRI- Empathic Concern		X			X	X	X		
CERQ - Reappraisal		X		X	X	X	X		
ML- Presence		X	X			X	X		
MAIA Self-regulation		X	X	X		X	X		
CEAS		X				X	X		
GACS		X					X		X

8 STATISTICAL ANALYSIS, SAMPLE SIZE AND POWER CALCULATIONS

8.1 DESIGN OVERVIEW

This is a two-arm parallel RCT with a wait-list controlled design in which the control group may pursue professional development activities as usual and will receive access to the intervention following the final study assessment nine-months post-randomization. We will enroll up to 4,000 participants within the recruitment window, which closes on May 21, 2023.

8.2 SAMPLE SIZE AND POWER ANALYSES

Our target sample size was informed in part by our agreements with State Ministers of Health, which requested a minimum number of participants from each participating state. Because of the large, planned sample size (up to 4,000), we conducted two power analyses. First, we conducted a sensitivity power analysis to determine the smallest detectable effect based on the lower bound of the expected intention-to-treat sample of 4,000. To ensure our power estimates were conservative, we based power calculations off of the Bonferroni corrected two-tailed p-value assuming that all primary and secondary outcomes in the parent trial share the same family-wise error 0.05 error rate. We plan to test a total of 9 primary and secondary outcomes for a Bonferroni corrected error rate of 0.006. For measures collected at all 10 assessments points, we assume:

- 0.80 power
- A correlation of $r = .50$ between the same measure at different assessments
- Error rate of 0.006

Under the above conditions, we will be able to detect a significant group by time interaction effect of Cohen's $d \geq 0.04$. For measures assessed at five of the timepoints, assuming the same conditions listed above, we will be able to detect between group effects of Cohen's $d \geq 0.05$.

We conducted a second power analysis to determine the size of the randomly selected biomarker and patient satisfaction report subsample required to detect a pre- to post-test change of $d \geq 0.15$. We selected $d \geq 0.15$ as our expected effect size because, in two meta-analyses on the topic of mindfulness-based interventions effects on inflammation, $d \geq 0.15$ was the smallest observed average effect size on the cytokines we will assay. We assume:

- 0.90 power
- A correlation of $r = .60$ between the same measure at different assessments
- Error rate of 0.005

In order to detect a statistically significant group by time interaction effect of Cohen's $d \geq 0.15$ on our measures of peripheral inflammation, we will require a subsample of 468 participants. Assuming 10% of selected participants do not provide a pre-test sample, we plan to randomly select 255 participants from each group (510 total) to participate in biomarker data collection.

8.3 INTERIM MONITORING

Interim monitoring will focus on survey completion, intervention engagement and safety. No interim efficacy analyses will be conducted.

8.4 STATISTICAL ANALYSIS PLAN

See https://osf.io/9g58j for preregistration of statistical plan, hypotheses and outcomes.

Primary and secondary outcomes will be analyzed using linear mixed effects models with time nested within participant. All randomized participants will be included (i.e., intention-to-treat analyses). Maximum likelihood estimation will be used for missing data. To help determine the best fit for modeling change over time, we will plot outcomes over time by group using loess regression. We will then quantitatively compare the Akaike Information Criterion (AIC) and Bayesian Information Criterion (BIC) of candidate models of linear change, loglinear change, polynomial change, and piecewise linear change in which change over the intervention period is modeled separately from change over follow-up. Primary effects of interest are groupXtime interactions from pre- to post, pre- to 3-month follow-up, and pre- to six month follow-up. We will control for gender and age. Models with and without social desirable responding will be compared and the best fitting model will be used.

Covariates may be centered to aid in model interpretation. Statistical significance will be set at a two-tailed alpha of $p<0.05$, false discovery rate corrected for the 9 inferential tests at post-test (2 primary, 7 secondary outcomes) and the three inferential tests at 6-month follow-up. In addition to statistical significance, magnitude of differences will be estimated using standardized mean differences on continuous outcomes and Odds Ratios on binary outcomes.

Before hypothesis testing, we will use the "careless" package in R to check for patterns of aberrant responding on the survey items. Should evidence for inattentive responding be observed, we will report results with and without affected data removed. Given the expected sample size, we do not expect outliers or high leverage points to be observed but we will use simulation methods to check models for these points. Should high influence points be observed, we will report models with and without them.

Should evidence for data not missing at random be observed (e.g., differential attrition between groups, inability to predict missingness based on observed variables) we will conduct pattern-mixture modeling.

We will examine moderation of intervention effects on primary and secondary outcomes by participants gender, age, employment type and location.

9 DATA MANAGMENT

Data will be collected for research purposes only. Data will be stored and handled according to agreements with State Minister's of Health, supervising review board in Mexico, and policies of the University of Wisconsin Madison.

9.1.1 DATA MONITORING PROCEDURES

The Mexico based implementation team and US based research team will meet weekly throughout the project to ensure data quality and proper data management and storage.

10 SAFETY AND ADVERSE EVENTS

10.1 DEFINTIONS

Adverse Event (AE): An AE is as any untoward medical occurrence in a participant and which does not necessarily have a causal relationship with a study treatment.

Serious Adverse Event (SAE): A SAE is any AE that:

- Results in death;
- Is life threatening, or places the participant at immediate risk of death from the event as it occurred;
- Requires or prolongs hospitalization;
- Causes persistent or significant disability or incapacity;
- Results in congenital anomalies or birth defects;
- Is another condition, which the investigators judge to represent significant hazards.

Unanticipated Problem: any incident, experience, or outcome that meets all of the following criteria:

- Unexpected, in terms of nature, severity, or frequency, given the research procedures that are described and the characteristics of the study population;
- Related or possibly related to participation in the research;
- Suggests that the research places participants or others at a greater risk of harm (including physical, psychological, economic, or social harm) than was previously known or recognized.

Adverse Event Reporting Period: The period from the initiation of study procedures to the end of the study treatment follow-up.

Preexisting Condition: A preexisting condition is one that is present at the time of providing the consent for the study. A preexisting condition is considered an adverse event if the frequency, intensity, or the character of the condition worsens during the study period.

11. ETHICAL AND REGULATORY CONSIDERATIONS

11.1 INFORMED CONSENT

11.2 INSTITUTIONAL REVIEW BOARD

Multiple review boards will provide oversight of this research. Mexico-based review boards provide primary oversight and the UW Madison IRB will oversee UW-Madison researcher's use

of data. All participants must provide informed consent before engaging in any study related activities.

11.3 RESPONSIBILITIES

Investigators conducting research are responsible for:

- Assurance that procedures are conducted in compliance with these guidelines.
- Receipt of appropriate ethics board approvals prior to onset of the research activities.
- Adherence to the procedures outlined in this document.

11.4 ASCERTAINMENT OF AE, UNANTICIPATED PROBLEMS, AND SAE

The PIs will record all reportable events with start dates occurring any time after informed consent is obtained until 7 (for non-serious AEs) or 30 days (for SAEs) after the last day of study participation. Unanticipated problems will be recorded in the data collection system throughout the study.

This trial involves a low-risk behavioral intervention. Nevertheless, we will monitor AE, UA and SAE through assessments (which ask about symptoms levels, and provide all participants with study staff they can call to report AE, UA or SAE. Adverse events will be recorded and reported to the PIs and ethics boards. An on-call clinician is available to support participants reporting any AE, UP or SAE. On a case by case basis, a participation in the study may be terminated should the AE, UP or SAE be deemed likely related to study activities.

11.5 SAFETY

11.5.1 Safety Personnel

In addition to study staff, an on call clinician is available for participants reporting AE, UP or SAE. Several of the intervention instructors are physicians and are able to provide emergency medical care if needed during an intervention session.

11.5.2 Reporting of unanticipated problems and SAE

A summary report of AEs, UP and SAE will be provided to oversight boards as needed and in accordance with regulatory body policies.

12 RISK TO HUMAN SUBJECTS

12.1 Protections against risk

Risk associated with the ISTH intervention is deemed minimal. However, AE, UP and SAE remain possible. We will therefore follow the procedures set forth above to monitor and respond to risk. In addition, there are potential risks associated with data collection and information management that we will mitigate by following struct data management procedures. Every effort will be made to inform the participant of this potential and minimize the risks as outlined

12.2 Potential benefits of the proposed research to human participants and others

There are few evidence-based programs to support the mental health and well-being of healthcare providers, who as a group report very high levels of distress. This study has the potential to identify an effective, scalable approach to promoting healthcare provider well-being.

12.3 Importance of the knowledge to be gained

It is likely that this study will provide novel information about the effectiveness of a scalable well-being training in healthcare providers as well as novel insight into the mechanisms through which such an intervention produces benefits.

13. STUDY DOCUMENTS

13.1 RETENTION OF RECORDS

All study documents will be retained for at least a period of five years following the completion

or discontinuation of this study.

14. PUBLICATION AND DISSEMINATION POLICY

PIs and collaborators will set rules for publication. The primary study preregistration will be published first.
