# Supplementary material for: Digital Well-Being Training With Health Care Professionals: A Randomized Clinical Trial
Source: JAMA Intern Med. 2025 Aug 18;185(10):1248–56. doi: 10.1001/jamainternmed.2025.3888 (PMC12362274; doi:10.1001/jamainternmed.2025.3888)
Supplement: Supplement 2. — eTable 1. Table of assessments eMethod 1. R statistical software packages used in the analyses reported in this research eMethod 2. Careless responding analyses eFigure 1. Trajectories of change on wellbeing skills by group, using observed data eFigure 2. Trajectories of change on burnout and gratitude by group, using observed data eTable 2. Primary and secondary outcomes at all timepoints by group eTable 3. Change in anxiety and depression symptoms by group eTable 4. Adverse events table eTable 5. Results of sensitivity analyses eTable 6. Descriptives statistics by group and status as an administrator eTable 7. Fit statistics from latent growth structural equation mediation models eTable 8. Description of socioeconomic levels coding [file jamainternmed-e253888-s002.pdf]

Supplemental Online Content

Hirshberg MJ, Davidson RJ, Arrisueño LV, et al; HCP-Well Study Group. Digital well-being training with health care professionals: a randomized clinical trial. *JAMA Intern Med.* Published online August 18, 2025. doi:10.1001/jamainternmed.2025.3888

eTable 1. Table of assessments

eMethod 1. R statistical software packages used in the analyses reported in this research

eMethod 2. Careless responding analyses

eFigure 1. Trajectories of change on wellbeing skills by group, using observed data

eFigure 2. Trajectories of change on burnout and gratitude by group, using observed data

eTable 2. Primary and secondary outcomes at all timepoints by group

eTable 3. Change in anxiety and depression symptoms by group

eTable 4. Adverse events table

eTable 5. Results of sensitivity analyses

eTable 6. Descriptives statistics by group and status as an administrator

eTable 7. Fit statistics from latent growth structural equation mediation models

eTable 8. Description of socioeconomic levels coding

This supplemental material has been provided by the authors to give readers additional information about their work.

© 2025 Hirshberg MJ et al. *JAMA Internal Medicine*.

eTable 1: Table of assessments

Event	Screen	Wk 0	Wk 1	Wk 3	Wk 5	Wk 8	Wk 13	Wk 25	Wk 37
Screening and Stratification Measures									
Informed Consent		X							
Demographics	X	X							
Primary and Secondary Outcomes									
PROMIS Anxiety & Depression, Perceived Stress		X	X	X	X	X	X	X	X
WHO-5		X	X	X	X	X	X	X	X
Healthy Minds Index		X	X		X	X	X		
GQ-6		X			X	X	X		
Maslach Burnout Inventory		X				X	X	X	X
Dried blood spots		X					X	X	
Tertiary Outcomes (Mechanisms)									
FFMQ		X		X		X	X		
IRI- Empathic Concern		X			X	X	X		
CERQ - Reappraisal		X		X	X	X	X		
ML- Presence		X	X			X	X		
MAIA Self-regulation		X	X	X		X	X		
CEAS		X				X	X		
GACS		X					X		X

Note. Baseline data were collected prior to random assignment. Wk stands for week. Wk 0 is the baseline assessment prior to random assignment. Wk 13 is the post-intervention primary study endpoint. Wk 37 is the six-month follow-up primary study endpoint. PROMIS is the Patient Reported Outcome Measurement Assessment System. WHO-5 is the World Health Organization 5. GQ-6 is the Gratitude Questionnaire 6. FFMQ is the Five Facet Mindfulness Questionnaire. IRI is the Interpersonal Reactivity Index. CERQ is the Cognitive Emotional Regulation Questionnaire. ML is the Meaning in Life Questionnaire. MAIA is the Multidimensional Assessment of Interoceptive Awareness. CEAS is the Compassionate Engagement and Action Scale. GACS is the Global Assessment of Character Skills.

© 2025 Hirshberg MJ et al. *JAMA Internal Medicine*.

eMethod 1: R statistical software packages used in the analyses reported in this research

1. Bates D, Maechler M, Bolker B, Walker S Fitting Linear Mixed-Effects Models Using lme4. *J. Stat. Softw*. 2015;67(1),1-48. doi:10.18637/jss.v067.i01.
2. Hothorn T, Bretz F, Westfall P. Simultaneous Inference in General Parametric Models. *Biom. J.*, 2028;50(3):346-363.
3. Kuznetsova A, Brockhoff PB, Christensen RHB. lmerTest Package: Tests in Linear Mixed Effects Models. 2017; *J. Stat. Softw*., 82(13),1-26. doi:10.18637/jss.v082.i13
4. Ma, W., Ye, X., Tu, F., & Hu, F. carat: An R Package for Covariate-Adaptive Randomization in Clinical Trials. *J. Stat. Softw*. 2023;*107*(2),1–47. https://doi.org/10.18637/jss.v107.i02
5. R Core Team. R: A language and environment for statistical computing. R Foundation for Statistical Computing, Vienna, Austria. URL https://www.R-project.org/. 2021
6. Revelle W. psych: Procedures for Psychological, Psychometric, and Personality Research. Northwestern University, Evanston, Illinois. R package version 2.4.3. 2024
7. Robitzsch, A., & Grund, S. miceadds: Some Additional Multiple Imputation Functions, Especially for 'mice'. R package version 3.17-44. 2024
8. van Buuren A. & Groothuis-Oudshoorn K. mice: Multivariate Imputation by Chained Equations *J. Stat. Softw*. 2011;45(3),1-67. DOI 10.18637/jss.v045.i03.
9. Wickham H, Averick M, Bryan J, et al. Welcome to the tidyverse. *JOSS*, 2019;4(43),1686. doi:10.21105/joss.01686
10. Yentes R.D., & Wilhelm F. careless: Procedures for computing indices of careless responding. R package version 1.2.2. 2023

© 2025 Hirshberg MJ et al. *JAMA Internal Medicine*.

eMethod 2: Careless responding analyses

We used the careless package in R to examine within participants and timepoints multiple indices of careless responding. First, we calculated *longstrings* (the number of items in a row answered the same way). Next, we split timepoints into item quartiles and calculated the standard deviation of responses across consecutive items within each quartile. Third, we identified *psychological synonyms*, or the number of items with a pairwise correlation above r=0.60 across the sample, and then computed the average correlation within participants across psychological synonyms. Fourth, we calculated Mahanalobis distance across all items within a timepoint to assess multivariate outliers. For the first the three indices, we used boxplots to identify participants more than 1.5 IQRs below the first quartile or above the third quartile. In the fourth, we looked for participants with a Mahalanobis D^2 with strong deviations from the sample distribution based on qqplots. Because no single index of careless responding can determine whether a participant's responses were careless, we cross-referenced participant ids across these four indices. Other than near neighbor approaches (i.e., longstrings and standard deviation of responses) we observed no consistent patterns of careless responding within any participant at any timepoint. Therefore, all observed data was retained.

© 2025 Hirshberg MJ et al. *JAMA Internal Medicine*.

eFigure 1: Trajectories of change on wellbeing skills by group, using observed data

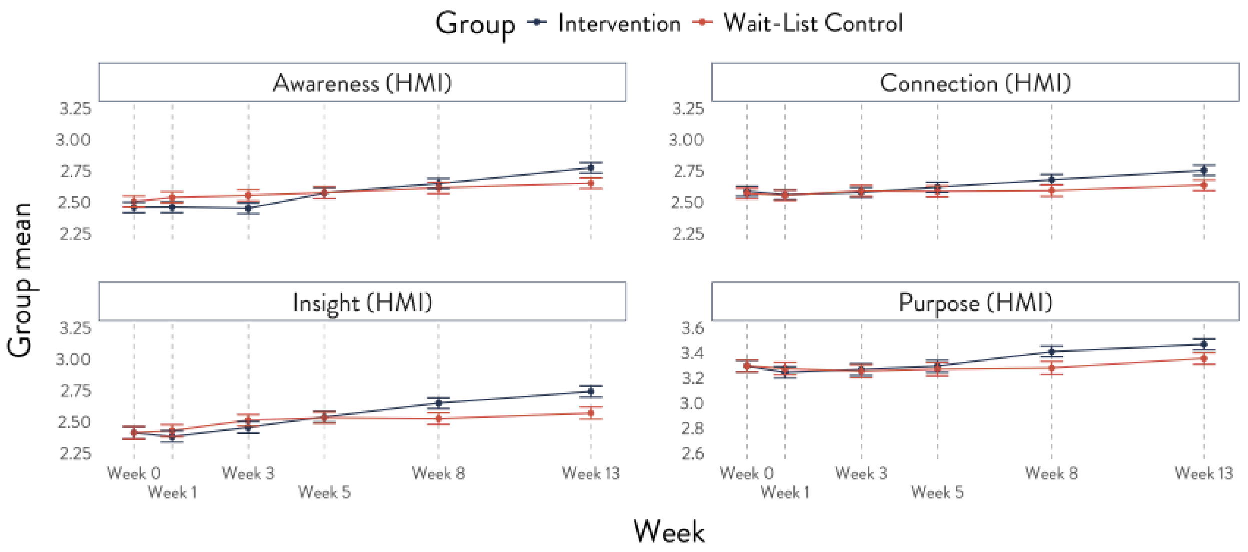

Note. Intervention is the Integrated Stress Toolbox for Healthcare Providers treatment arm. All plots are observed data at the timepoints assessed. Errors bars are 95% confidence intervals (standard error of the mean). Week corresponds to the number of weeks post randomization that the assessment occurred. Week 13 is the post-intervention primary endpoint. HMI stands for Healthy Minds Index.

© 2025 Hirshberg MJ et al. *JAMA Internal Medicine*.

eFigure2: Trajectories of change on burnout and gratitude by group, using observed data

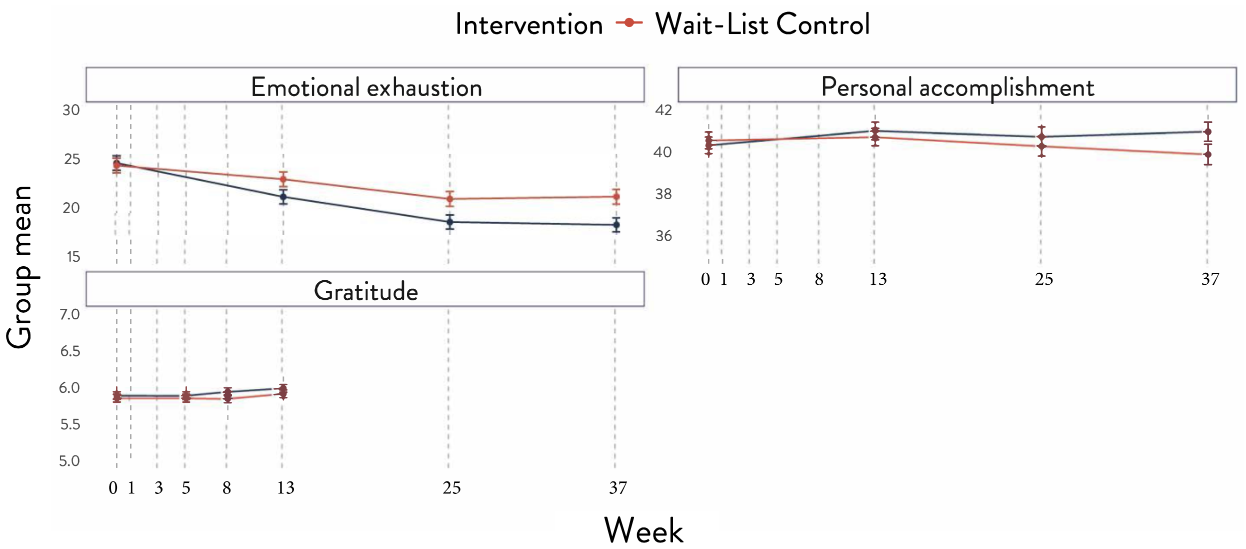

Note. Intervention is the Integrated Stress Toolbox for Healthcare Providers treatment arm. All plots are observed data at the timepoints assessed. Errors bars are 95% confidence intervals (standard error of the mean). Week corresponds to the number of weeks post randomization that the assessment occurred. Week 13 is the post-intervention primary endpoint. Week 37 is the follow-up primary endpoint.

© 2025 Hirshberg MJ et al. *JAMA Internal Medicine*.

eTable 2: Primary and secondary outcomes at all timepoints by group

Week (T)	Intervention n/N (%)	Intervention Mean (SD)	Wait-List Control n/N (%)	Wait-List Control Mean (SD)	Mean difference (95% CI)	Adjusted mean difference (95% CI)	Effect size (Cohen's d, 95% CI)	*P* value
**Distress: Z-scored and aggregated Perceived Stress Scale, PROMIS Anxiety, and PROMIS Depression scales**								
Baseline	1157/1157 (100%)	0.01 (0.89)	1158/1158 (100%)	0 (0.94)	0.01 (-0.06 to 0.08)			
Week 1	993/1157 (86%)	0.01 (0.88)	1033/1158 (89%)	-0.07 (0.93)	0.07 (-0.01 to 0.15)			
Week 3	963/1157 (83%)	-0.14 (0.88)	1014/1158 (88%)	-0.18 (0.98)	0.05 (-0.03 to 0.13)			
Week 5	845/1157 (73%)	-0.26 (0.87)	947/1158 (82%)	-0.21 (0.99)	-0.05 (-0.14 to 0.04)			
Week 8	897/1157 (78%)	-0.37 (0.87)	964/1158 (83%)	-0.23 (0.99)	-0.14 (-0.22 to -0.06)			
Week 13	819/1157 (71%)	-0.52 (0.84)	886/1158 (77%)	-0.28 (0.95)	-0.24 (-0.33 to -0.15)	-0.22 (-0.28 to - 0.15)	-0.24 (-0.31 to -0.17)	<0.001
Week 25	746/1157 (64%)	-0.52 (0.86)	783/1158 (68%)	-0.29 (0.95)	-0.24 (-0.33 to -0.15)			
Week 37	693/1157 (60%)	-0.55 (0.84)	767/1158 (66%)	-0.25 (0.94)	-0.30 (-0.39 to -0.21)	-0.33 (-0.41 to - 0.24)	-0.36 (-0.44 to -0.27)	<0.001
**WHO-5 Wellbeing (possible range 5 – 25)**								
Baseline	1157/1157 (100%)	14.86 (4.89)	1158/1158 (100%)	14.84 (4.98)	0.02 (-0.38 to 0.42)			
Week 1	999/1157 (86%)	14.8 (4.68)	1042/1158 (90%)	14.99 (4.83)	-0.19 (-0.6 to 0.22)			
Week 3	968/1157 (84%)	15.99 (4.75)	1018/1158 (88%)	15.78 (5.01)	0.2 (-0.23 to 0.63)			
Week 5	857/1157 (74%)	16.81 (4.62)	956/1158 (83%)	16.17 (5.1)	0.64 (0.19 to 1.09)			
Week 8	902/1157 (78%)	17.12 (4.42)	966/1158 (83%)	16.34 (5.12)	0.78 (0.35 to 1.21)			
Week 13	821/1157 (71%)	18.02 (4.46)	887/1158 (77%)	16.54 (4.92)	1.47 (1.02 to 1.92)	1.30 (0.93 to 1.68)	0.27 (0.19 to 0.34)	<0.001
Week 25	755/1157 (65%)	17.93 (4.48)	792/1158 (68%)	16.43 (4.93)	1.5 (1.03 to 1.97)			
Week 37	697/1157 (60%)	18.18 (4.14)	768/1158 (66%)	16.03 (5.08)	2.15 (1.68 to 2.62)	2.07 (1.97 to 2.18)	0.42 (0.34 to 0.5)	<0.001
**Healthy Minds Index Awareness (possible range 1 – 5)**								
Baseline	1157/1157 (100%)	2.46 (0.73)	1158/1158 (100%)	2.5 (0.78)	-0.05 (-0.11 to 0.01)			
Week 1	990/1157 (86%)	2.46 (0.75)	1030/1158 (89%)	2.54 (0.76)	-0.08 (-0.15 to -0.01)			
Week 3	962/1157 (83%)	2.45 (0.75)	1012/1158 (87%)	2.55 (0.77)	-0.1 (-0.17 to -0.03)			
Week 5	842/1157 (73%)	2.57 (0.74)	944/1158 (82%)	2.58 (0.78)	0 (-0.07 to 0.07)			
Week 8	896/1157 (77%)	2.65 (0.71)	962/1158 (83%)	2.61 (0.78)	0.03 (-0.04 to 0.1)			
Week 13	817/1157 (71%)	2.77 (0.72)	881/1158 (76%)	2.65 (0.77)	0.12 (0.05 to 0.19)	0.15 (0.10 to 0.21)	0.20 (0.13 to 0.28)	<0.001

© 2025 Hirshberg MJ et al. *JAMA Internal Medicine*.

**Healthy Minds Index Connection (possible range 1 – 5)**								
Baseline	1157/1157 (100%)	2.59 (0.66)	1158/1158 (100%)	2.57 (0.7)	0.02 (-0.04 to 0.08)			
Week 1	990/1157 (86%)	2.56 (0.66)	1030/1158 (89%)	2.55 (0.71)	0.01 (-0.05 to 0.07)			
Week 3	962/1157 (83%)	2.58 (0.68)	1012/1158 (87%)	2.59 (0.74)	-0.01 (-0.07 to 0.05)			
Week 5	842/1157 (73%)	2.62 (0.72)	944/1158 (82%)	2.58 (0.74)	0.03 (-0.04 to 0.1)			
Week 8	896/1157 (77%)	2.68 (0.74)	962/1158 (83%)	2.59 (0.77)	0.09 (0.02 to 0.16)			
Week 13	817/1157 (71%)	2.75 (0.71)	881/1158 (76%)	2.63 (0.75)	0.12 (0.05 to 0.19)	0.09 (0.04 to 0.15)	0.13 (0.05 to 0.22)	0.0014
**Healthy Minds Index Insight (possible range 1 – 5)**								
Baseline	1157/1157 (100%)	2.41 (0.83)	1158/1158 (100%)	2.41 (0.85)	0 (-0.07 to 0.07)			
Week 1	990/1157 (86%)	2.38 (0.78)	1030/1158 (89%)	2.43 (0.81)	-0.05 (-0.12 to 0.02)			
Week 3	962/1157 (83%)	2.45 (0.78)	1012/1158 (87%)	2.51 (0.8)	-0.06 (-0.13 to 0.01)			
Week 5	842/1157 (73%)	2.54 (0.77)	944/1158 (82%)	2.53 (0.8)	0.01 (-0.06 to 0.08)			
Week 8	896/1157 (77%)	2.65 (0.76)	962/1158 (83%)	2.52 (0.82)	0.12 (0.05 to 0.19)			
Week 13	817/1157 (71%)	2.74 (0.76)	881/1158 (76%)	2.57 (0.83)	0.17 (0.09 to 0.25)	0.19 (0.12 to 0.26)	0.23 (0.14 to 0.31)	<0.001
**Healthy Minds Index Purpose (possible range 1 – 5)**								
Baseline	1157/1157 (100%)	3.29 (0.79)	1158/1158 (100%)	3.29 (0.82)	0 (-0.07 to 0.07)			
Week 1	990/1157 (86%)	3.24 (0.77)	1030/1158 (89%)	3.27 (0.84)	-0.03 (-0.1 to 0.04)			
Week 3	962/1157 (83%)	3.27 (0.8)	1012/1158 (87%)	3.25 (0.87)	0.01 (-0.06 to 0.08)			
Week 5	842/1157 (73%)	3.29 (0.82)	944/1158 (82%)	3.27 (0.89)	0.02 (-0.06 to 0.1)			
Week 8	896/1157 (77%)	3.41 (0.74)	962/1158 (83%)	3.28 (0.87)	0.13 (0.06 to 0.2)			
Week 13	817/1157 (71%)	3.47 (0.74)	881/1158 (76%)	3.35 (0.82)	0.11 (0.04 to 0.18)	0.13 (0.07 to 0.19)	0.16 (0.09 to 0.23)	<0.001
**GQ-6 Gratitude (possible range 1 – 7)**								
Baseline	1157/1157 (100%)	5.89 (0.88)	1158/1158 (100%)	5.85 (0.94)	0.04 (-0.03 to 0.11)			
Week 5	837/1157 (72%)	5.88 (0.93)	936/1158 (81%)	5.85 (0.94)	0.03 (-0.06 to 0.12)			
Week 8	893/1157 (77%)	5.94 (0.95)	961/1158 (83%)	5.84 (0.97)	0.09 (0 to 0.18)			
Week 13	817/1157 (71%)	5.99 (0.91)	875/1158 (76%)	5.91 (0.93)	0.08 (-0.01 to 0.17)	0.01 (-0.04 to 0.02)	0.01 (-0.04 to 0.02)	0.5280
**Maslach Burnout Inventory Emotional exhaustion (possible range 0 – 54)**								
Baseline	1157/1157 (100%)	24.53 (12.77)	1158/1158 (100%)	24.27 (12.93)	0.26 (-0.79 to 1.31)			
Week 13	814/1157 (70%)	21.09 (12.39)	872/1158 (75%)	22.88 (12.89)	-1.79 (-3 to -0.58)	-1.59 (-2.52 to -0.66)	-0.12 (-0.20 to -0.05)	<0.001

© 2025 Hirshberg MJ et al. *JAMA Internal Medicine*.

Week 25	741/1157 (64%)	18.53 (12.34)	778/1158 (67%)	20.87 (13.07)	-2.34 (-3.62 to -1.06)			
Week 37	688/1157 (59%)	18.25 (12.18)	767/1158 (66%)	21.11 (13.07)	-2.86 (-4.16 to -1.56)	-2.45 (-2.56 to -2.34)	-0.19 (-0.27 to -0.11)	<0.001
**Maslach Burnout Inventory Personal accomplishment (possible range 0 – 48)**								
Baseline	1157/1157 (100%)	40.3 (6.93)	1158/1158 (100%)	40.53 (6.93)	-0.23 (-0.79 to 0.33)			
Week 13	814/1157 (70%)	40.98 (7.29)	872/1158 (75%)	40.69 (7.23)	0.29 (-0.4 to 0.98)	0.47 (-0.15 to 1.09)	0.07 (-0.02 to 0.16)	0.1529
Week 25	741/1157 (64%)	40.71 (8.13)	778/1158 (67%)	40.26 (8.14)	0.45 (-0.37 to 1.27)			
Week 37	688/1157 (59%)	40.95 (7.85)	767/1158 (66%)	39.87 (8.42)	1.08 (0.24 to 1.92)	1.22 (1.13 to 1.31)	0.18 (0.09 to 0.26)	<0.001

Note. Intervention is the Integrated Stress Toolbox for Healthcare Providers group. All *P*-values are false discovery rate corrected.

© 2025 Hirshberg MJ et al. *JAMA Internal Medicine*.

eTable 3: Change In anxiety and depression symptoms by group

Week	Intervention n/N (%)	Intervention Mean (SD)	Intervention Change	Wait-List Control n/N (%)	Wait-List Control Mean (SD)	Wait-List Control Change
**PROMIS Anxiety**						
Baseline	1157/1157 (100%)	56.31 (7.69)		1158/1158 (100%)	56.31 (8.12)	
Week 13	820/1157 (71%)	51.51 (7.79)	$\Delta= 4.6$	887/1158 (77%)	53.86 (8.72)	$\Delta= 2.45$
Week 37	695/1157 (60%)	51.85 (7.91)	$\Delta= 4.47$	768/1158 (66%)	53.91 (8.52)	$\Delta= 2.40$
**PROMIS Depression**						
Baseline	1157/1157 (100%)	50.80 (8.61)		1158/1158 (100%)	50.49 (9.17)	
Week 13	819/1157 (71%)	46.72 (8.25)	$\Delta= 4.08$	887/1158 (77%)	48.65 (9.00)	$\Delta= 1.84$
Week 37	694/1157 (60%)	46.34 (8.09)	$\Delta= 4.46$	768/1158 (66%)	49.13 (8.92)	$\Delta= 1.36$

Note. Intervention is the Integrated Stress Toolbox for Healthcare Providers group. PROMIS Anxiety and Depression 8-item version 2 forms were used. Change is the difference (Δ) between baseline and the 13 week post-intervention assessment and between baseline and 37 week follow-up assessment, respectively. For PROMIS anxiety a change of +/- 3 and for PROMIS depression a change of +/- 3 to 4 is considered the minimal clinically important difference threshold.

© 2025 Hirshberg MJ et al. *JAMA Internal Medicine*.

eTable 4: Adverse events table

	Intervention		Control group	
	n/ adverse events N (%)	n/ group N (%)	n/ adverse events N (%)	n/ group N (%)
**Study unrelated adverse events**	**4**		**1**	
Death (hypertension)	1/4(25·0%)	1/1157 (<0·1%)	0	0
Sickness (eye surgery)	1/4 (25·0%)	1/1157 (<0·1%)	0	0
Mental health	2/4 (50·0%)	2/1157 (<0·2%)	1/1 (100%)	1/1157 (<0·1%)
**Study related adverse events**	**2**		**1**	
Stress and overwhelm	1/2 (50·0%)	1/1157 (<0·1%)	1/1(100%)	1/1157 (<0·1%)
Suicidal ideation (relapse)	1/2 (50·0%)	1/1157 (<0·1%)	0	0

Note. The mental health events in the intervention group deemed study unrelated (n=2) were due to depression following a failed medical procedure (n=1) and sadness due to a romantic partner breakup (n=1). Study unrelated mental health in the control group (n=1) was due to losing a patient (n=1).

© 2025 Hirshberg MJ et al. *JAMA Internal Medicine*.

eTable 5: Results of sensitivity analyses

	Missing data assumed to be 10% worse than imputed		Missing data assumed to be 20% worse than imputed	
**Week**	**Adjusted mean difference (95% CI)**	**Effect size (Cohen's d, 95% CI)**	**Adjusted mean difference (95% CI)**	**Effect size (Cohen's d, 95% CI)**
Distress				
13	-0·21 (-0·29 to -0·13)	-0·21 (-0·31 to -0·15)	-0·22 (-0·28 to -0·13)	-0·22 (-0·34 to -0·11)
37	-0·26 (-0·42 to -0·13)	-0·26 (-0·35 to -0·18)	-0·26 (-0·42 to -0·09)	-0·26 (-0·38 to -0·14)
Wellbeing				
13	1·02 (0·67 to 1·37)	0·21 (0·11 to 0·31)	0·95 (0·57 to 1·31)	0·19 (0·11 to 0·27)
37	1·44 (0·63 to 2·26)	0·29 (0·20 to 0·40)	1·41 (0·58 to 2·21)	0·29 (0·21 to 0·37)
Awareness				
13	0·13 (0·06 to 0·19)	0·17 (0·10 to 0·25)	0·11 (0·05 to 0·17)	0·15 (0·09 to 0·21)
Connection				
13	0·06 (0·00 to 0·12)	0·09 (-0·01 to 0·19)	0·05 (-0·02 to 0·11)	0·07 (-0·01 to 0·15)
Insight				
13	0·13 (0·06 to 0·21)	0·16 (0·06 to 0·26)	0·12 (0·04 to 0·19)	0·14 (0·06 to 0·23)
Purpose				
13	0·09 (0·02 to 0·16)	0·11 (0·02 to 0·20)	0·07 (0·02 to 0·14)	0·09 (0·00 to 0·17)
Gratitude				
13	0·01 (-0·07 to 0·09)	0·01 (-0·09 to 0·10)	-0·13 (-0·31 to 0·25)	-0·01 (-0·09 to 0·06)
Emotional exhaustion				
13	-1·34 (-2·56 to -0·12)	-0·10 (-0·23 to 0·03)	-1·24 (-2·53 to -0·05)	-0·10 (-0·24 to 0·04)
37	-1·85 (-4·53 to -0·84)	-0·14 (-0·27 to -0·01)	-1·73 (-4·58 to 1·13)	-0·13 (-0·27 to 0·00)
Personal accomplishment				
13	0·21 (-0·52 to 0·93)	0·03 (-0·08 to 0·14)	0·04 (-0·69 to 0·78)	0·01 (-0·09 to 0·10)
37	0·61 (-0·94 to 2·16)	0·09 (-0·02 to 0·20)	0·37 (-1·15 to 1·90)	0·05 (-0·04 to 0·15)

© 2025 Hirshberg MJ et al. *JAMA Internal Medicine*.

eTable 6: Descriptives statistics by group and status as an administrator

Outcome	Time	Intervention (*n*=1157) Provider Mean (*SD*)	Intervention (*n*=1157) Administrator Mean (*SD*)	Control (*n*=1158) Provider Mean (*SD*)	Control (*n*=1158) Administrator Mean (*SD*)
No.	Baseline	*n*=1061	*n*=96	*n*=1071	*n*=87
Distress	Baseline	0.01 (0.90)	-0.02 (0.79)	0 (0.95)	-0.02 (0.89)
Distress	Week 13	-0.52 (0.85)	-0.51 (0.80)	-0.28 (0.96)	-0.28 (0.92)
Distress	Week 37	-0.57 (0.82)	-0.36 (0.96)	-0.25 (0.94)	-0.27 (0.96)
Wellbeing	Baseline	14.90 (4.88)	14.43 (5.00)	14.86 (5.00)	14.51 (4.72)
Wellbeing	Week 13	18.01 (4.46)	18.14 (4.5)	16.56 (4.92)	16.4 (4.98)
Wellbeing	Week 37	18.31 (4.03)	17.23 (4.8)	15.93 (5.12)	16.96 (4.71)
Emotional exhaustion	Baseline	24.79 (12.78)	21.61 (12.32)	24.5 (12.87)	21.5 (13.52)
Emotional exhaustion	Week 13	21.26 (12.48)	19.54 (11.44)	23.08 (12.92)	20.98 (12.52)
Emotional exhaustion	Week 37	18.21 (12.15)	18.51 (12.47)	21.36 (12.93)	18.94 (14.10)
Personal accomplishment	Baseline	40.62 (6.66)	36.82 (8.79)	40.72 (6.76)	38.14 (8.48)
Personal accomplishment	Week 13	41.28 (7.01)	38.27 (9.14)	40.96 (7.13)	38.16 (7.78)
Personal accomplishment	Week 37	41.42 (7.38)	37.39 (10.1)	40.15 (8.33)	37.44 (8.85)
Awareness	Baseline	2.46 (0.73)	2.43 (0.74)	2.49 (0.78)	2.65 (0.66)
Awareness	Week 13	2.78 (0.72)	2.67 (0.7)	2.65 (0.77)	2.69 (0.74)
Connection	Baseline	2.58 (0.67)	2.7 (0.57)	2.56 (0.7)	2.67 (0.63)
Connection	Week 13	2.75 (0.72)	2.78 (0.6)	2.63 (0.75)	2.64 (0.74)
Insight	Baseline	2.42 (0.82)	2.37 (0.91)	2.42 (0.85)	2.27 (0.82)
Insight	Week 13	2.75 (0.76)	2.7 (0.78)	2.59 (0.82)	2.37 (0.95)
Purpose	Baseline	3.29 (0.79)	3.27 (0.71)	3.30 (0.82)	3.22 (0.84)
Purpose	Week 13	3.48 (0.72)	3.34 (0.89)	3.36 (0.82)	3.32 (0.85)
Gratitude	Baseline	5.89 (0.88)	5.91 (0.80)	5.85 (0.94)	5.90 (0.94)
Gratitude	Week 13	5.99 (0.91)	5.98 (0.85)	5.90 (0.93)	5.96 (0.84)

Note. Provider is patient facing healthcare providers. Administrators are healthcare administrators. Baseline data was collected prior to random assignment. Week 13 is the post-intervention primary endpoint. Week 37 is the six month follow-up primary endpoint.

© 2025 Hirshberg MJ et al. *JAMA Internal Medicine*.

eTable 7: Fit statistics from latent growth structural equation mediation models

	Distress				Wellbeing			
Model	**CFI**	**TLI**	**RMSEA**	**SRMR**	**CFI**	**TLI**	**RMSEA**	**SRMR**
**Awareness**	0·981	0·980	0·038 90% CI[0·03, 0·04]	0·037	0·984	0·983	0·033 90% CI[0·03, 0·04]	0·040
**Connection**	0·982	0·981	0·037 90% CI[0·03, 0·04]	0·052	0·989	0·988	0·028 90% CI[0·02, 0·04]	0·044
**Insight**	0·979	0·972	0·040 90% CI[0·03, 0·05]	0·063	0·980	0·979	0·039 90% CI[0·03, 0·04]	0·032
**Purpose**	0·972	0·971	0·042 90% CI[0·04, 0·05]	0·037	0·982	0·981	0·036 90% CI[0·03, 0·04]	0·065

Note· CFI= TLI = Tucker-Lewis Index· RMSEA = Root mean square error of approximation· SRMR = Standardized root mean square residual.

© 2025 Hirshberg MJ et al. *JAMA Internal Medicine*.

eTable 8: Description of socioeconomic levels coding

Socioeconomic level	Description
**A/B**	**Characteristics:** High purchasing power and indicators of a high standard of living.<br>• **Education:** 78% of household heads have at least postgraduate studies, reflecting strong investment in education.<br>• **Housing:** 72% of homes have at least three bedrooms, providing ample space for family. Additionally, 70% own at least two cars, reflecting significant mobility and convenience.<br>• **Connectivity:** Nearly all (99%) households have internet access.<br>• **Technology:** 32% of households own at least two computers, indicating active adoption of technology and access to digital tools.
**C+**	**Characteristics:** Intermediate standard of living with moderate comfort levels.<br>• **Education:** 69% of household heads have at least a high school education, indicating a mid-level educational attainment.<br>• **Housing:** 53% of homes have at least three bedrooms, providing adequate family space. Additionally, 35% own at least two cars, reflecting reasonable mobility and convenience.<br>• **Connectivity:** 98% of households have internet access.<br>• **Technology:** 13% of households own at least two computers, reflecting moderate technology adoption.<br>• **Spending:** About 32% of income is spent on food.
**C**	**Characteristics:** Basic to intermediate standard of living with adequate resources.<br>• **Education:** 79% of household heads have at least a secondary school education, reflecting basic or intermediate educational attainment.<br>• **Housing:** 41% of homes have at least three bedrooms, providing sufficient space for families.<br>• **Connectivity:** 92% of households have fixed internet access, critical for communication, work, and access to information.<br>• **Technology:** 6% of households own at least two computers, indicating basic technology adoption.<br>• **Spending:** 36% of income is allocated to food, and 18% of households own at least two cars.
**C-**	**Characteristics:** Limited resources and basic living conditions.<br>• **Education:** 67% of household heads have secondary education at most, reflecting basic educational attainment.<br>• **Housing:** 78% of households live in homes with one or two bedrooms, indicating limited space compared to higher levels.<br>• **Connectivity:** 80% of households have fixed internet access, critical for communication and information.<br>• **Technology:** 28% of households own at least one computer, reflecting basic technology adoption.<br>• **Spending:** 38% of income goes to food, and 20% to transportation.
**D+**	**Characteristics:** Basic living conditions with modest access to resources.<br>• **Education:** 76% of household heads have secondary education at most, reflecting basic educational attainment.<br>• **Housing:** 80% of households live in homes with up to two bedrooms, indicating limited space.<br>• **Connectivity:** 57% of households have fixed internet access, essential for communication and information.<br>• **Technology:** 16% of households own at least one computer, indicating minimal technology adoption.<br>• **Spending:** 42% of income is allocated to food, and 18% to transportation.

© 2025 Hirshberg MJ et al. *JAMA Internal Medicine*.

**D**	**Characteristics:** Limited access to resources and constrained living conditions.<br>• **Education:** 55% of household heads have primary education, reflecting basic educational attainment.<br>• **Housing:** 87% of households live in homes with one or two bedrooms, indicating limited space.<br>• **Connectivity:** Only 17% of households have fixed internet access, indicating low connectivity.<br>• **Technology:** 4.1% of households own at least one computer, reflecting minimal technology adoption.<br>• **Spending:** 46% of income is allocated to food, prioritizing basic needs.
**E**	**Characteristics:** Severely constrained living conditions and access to resources.<br>• **Education:** 83% of household heads have primary education at most, reflecting basic educational attainment.<br>• **Housing:** 70% of homes have only one bedroom, reflecting extremely limited space. 82% lack a full bathroom.<br>• **Connectivity:** 0.3% of households have internet access.<br>• **Technology:** Computers are virtually nonexistent in these households.<br>• **Spending:** 52% of income is allocated to food, and only 3% to education, reflecting prioritization of basic survival needs.

© 2025 Hirshberg MJ et al. *JAMA Internal Medicine*.
