# Supplementary material for: Digital Well-Being Training With Health Care Professionals: A Randomized Clinical Trial
Source: JAMA Intern Med. 2025 Aug 18;185(10):1248–56. doi: 10.1001/jamainternmed.2025.3888 (PMC12362274; doi:10.1001/jamainternmed.2025.3888)
Supplement: Supplement 3. — Nonauthor Collaborators [file jamainternmed-e253888-s003.pdf]

Supplemental Online Content: Nonauthor Collaborators
*First name, last name, and suffix (if applicable) are required and will appear in PubMed.

*Group Name(s): HCP-Well Study Group							
***First Name and Middle Initial(s)**	***Last Name**	***Suffix (eg, Jr, III)**	Academic Degrees	Institution	Location (city, state/province, country)	Role or Contribution, eg, chair, principal investigator	Group (if more than 1 Group listed in the byline) and/or Subgroup (eg, Steering Committee)
Alma R	Marroquín Escamilla		PhD	Secretaría de Salud del Estado y Servicios	Nuevo León, México	Senior Program Officer	Government partners
M Mirthala	Cavazos Parra		PhD	Secretaría de Salud del Estado y Servicios	Nuevo León, México	Senior Program Officer	Government partners
Roberto	Montes de Oca Luna		PhD	Secretaría de Salud del Estado y Servicios	Nuevo León, México	Program Officer	Government partners
Edgar P	Rodríguez Vidales		PhD	Secretaría de Salud del Estado y Servicios	Nuevo León, México	Program Officer	Government partners
Virginia G	Peña López		MD	Secretaría de Salud del Estado y Servicios	Nuevo León, México	Program Coordinator	Government partners
Sandra	Rodriguez Aguayo		MD	Secretaría de Salud del Estado y Servicios	Nuevo León, México	Program Coordinator	Government partners
Eliud F	Aguirre Vázquez		MD	Servicios de Salud de Coahuila de Zarago	Coahuila de Zaragoza, México	Senior Program Officer	Government partners
Raúl	Rodríguez Sánchez		MD	Servicios de Salud de Coahuila de Zarago	Coahuila de Zaragoza, México	Senior Program Officer	Government partners
Angela M	González García		MSc	Servicios de Salud de Coahuila de Zarago	Coahuila de Zaragoza, México	Program Officer	Government partners
Liliana G	Martell Valdez		EdM	Servicios de Salud de Coahuila de Zarago	Coahuila de Zaragoza, México	Program Coordinator	Government partners
Liliana A	Montejo León		MSPH	Órgano Público Descentralizado de los Se	Campeche, México	Senior Program Officer	Government partners
Josefa	Castillo Avendaño		MACH	Secretaría de Salud e Instituto de Servici	Campeche, México	Senior Program Officer	Government partners
Salvador	Chacón Ramírez		MBBS	Secretaría de Salud e Instituto de Servici	Campeche, México	Senior Program Officer	Government partners
Karla DA	De la Cruz Góngora		MD	Secretaría de Salud e Instituto de Servici	Campeche, México	Senior Program Officer	Government partners
Sheyla A	López Dado		MSP	Secretaría de Salud e Instituto de Servici	Campeche, México	Program Coordinator	Government partners
Karina A	Muñoz Canche		MCE	Secretaría de Salud e Instituto de Servici	Campeche, México	Program Coordinator	Government partners
Corazón	Moreno Mex		MBBS	Secretaría de Salud e Instituto de Servici	Campeche, México	Program Coordinator	Government partners
Mizueth J	Peralta Orduño		MBBS	Secretaría de Salud e Instituto de Servici	Campeche, México	Program Coordinator	Government partners
Alma L	Velasco Hernández		MSc	Servicios de Salud de Oaxaca	Oaxaca, México	Senior Program Officer	Government partners
Efrén E	Jarquin González		MSc	Servicios de Salud de Oaxaca	Oaxaca, México	Senior Program Officer	Government partners
Alejandro	López Bautista		MHSA	Servicios de Salud de Oaxaca	Oaxaca, México	Program Officer	Government partners
Ivett	Caballero López		MPH	Servicios de Salud de Oaxaca	Oaxaca, México	Program Officer	Government partners
Christell	Gómez Navarro		BA	Servicios de Salud de Oaxaca	Oaxaca, México	Program Officer	Government partners
Karla	Cruz Martínez		MPH	Servicios de Salud de Oaxaca	Oaxaca, México	Program Officer	Government partners
Gie'rini	Jiménez Sánchez		BSc	Servicios de Salud de Oaxaca	Oaxaca, México	Program Coordinator	Government partners
Gabriela E	Jiménez Valladolid		MBBS	Servicios de Salud de Oaxaca	Oaxaca, México	Program Coordinator	Government partners
M Martina	Pérez Réndon		PhD	Servicios de Salud del Estado de Querétа	Querétaro, México	Senior Program Officer	Government partners
Francisco J	Rivera Pesquera		PhD	Servicios de Salud del Estado de Querétа	Querétaro, México	Senior Program Officer	Government partners
Martha EM	Patiño Aboytes		MD	Servicios de Salud del Estado de Querétа	Querétaro, México	Program Officer	Government partners

Supplemental Online Content: Nonauthor Collaborators
*First name, last name, and suffix (if applicable) are required and will appear in PubMed.

***First Name and Middle Initial(s)**	***Last Name**	***Suffix (eg, Jr, III)**	Academic Degrees	Institution	Location (city, state/province, country)	Role or Contribution, eg, chair, principal investigator	Group (if more than 1 Group listed in the byline) and/or Subgroup (eg, Steering Committee)
Nora A	Castro Montes		PhD	Servicios de Salud del Estado de Queréta	Querétaro, México	Program Officer	Government partners
Ivette	Mata Maqueda		MD	Servicios de Salud del Estado de Queréta	Querétaro, México	Program Coordinator	Government partners
Gabriela C	Nucamendi Cervantes		PhD	Órgano Público Descentralizado de los Se	Sonora, México	Senior Program Officer	Government partners
Sara M	Córdova Manrique		MD	Órgano Público Descentralizado de los Se	Sonora, México	Program Officer	Government partners
Luis G	Beltrán Galindo		PhD	Órgano Público Descentralizado de los Se	Sonora, México	Program Coordinator	Government partners
José L	Alomía		PhD	Servicios de Salud de Sonora	Sonora, México	Senior Program Officer	Government partners
Eva	Moncada García		MD	Servicios de Salud de Sonora	Sonora, México	Program Officer	Government partners
Vania Z	Griego Castillo		MSc	Servicios de Salud de Sonora	Sonora, México	Program Coordinator	Government partners
Mariana	Gil Veloz		MD	AtentaMente Consultores AC	Guanajuato, México	Program Officer	Implementing organization
Guillermo E	Aréchiga Ornelas		MD	AtentaMente Consultores AC	Jalisco, México	Program Officer	Implementing organization
Daniela N	Labra Cardero		BSc	AtentaMente Consultores AC	Mexico City, México	Steering Committee	Implementing organization
Ana E	Fernandez Silva		BA	AtentaMente Consultores AC	Jalisco, México	Academic Coordinator	Implementing organization
Patricia A	Peña Aguirra		MBA	AtentaMente Consultores AC	Coahuila de Zaragoza, México	Program Officer	Implementing organization
Karla	Garza García		EdM	AtentaMente Consultores AC	Coahuila de Zaragoza, México	Program Officer	Implementing organization
Irene	Morales Zúñiga		BSEd	AtentaMente Consultores AC	Cauca, Colombia	Program Officer	Implementing organization
Adriana A	Lejarza Pérez		MPP	AtentaMente Consultores AC	Mexico City	Program Officer	Implementing organization
L Amanda	Zamora Sánchez		BSc	AtentaMente Consultores AC	Mexico City	Program Officer	Implementing organization
Jorge J	Tafich Acuña		BSc	AtentaMente Consultores AC	Mexico City	Program Officer	Implementing organization
Adrián M	Aguilar Orta		MSc	AtentaMente Consultores AC	Mexico City	Data Analyst	Implementing organization
Cesar	Carrera Vasquez		BSc	AtentaMente Consultores AC	Lima, Perú	Data Analyst	Implementing organization
