# Supplementary material for: Digital Well-Being Training With Health Care Professionals: A Randomized Clinical Trial
Source: JAMA Intern Med. 2025 Aug 18;185(10):1248–56. doi: 10.1001/jamainternmed.2025.3888 (PMC12362274; doi:10.1001/jamainternmed.2025.3888)
Supplement: Supplement 4. — Data Sharing Statement [file jamainternmed-e253888-s004.pdf]

Data Sharing Statement

Hirshberg. Digital Well-Being Training With Health Care Professionals. *JAMA Intern Med*. Published August 18, 2025. doi:10.1001/jamainternmed.2025.3888

Data
Additional Information: Clinicaltrials.gov (NCT05767970), https://clinicaltrials.gov/study/NCT05767970
Data available: Yes
Data types: Deidentified participant data
How to access data: Data will posted at https://osf.io/28myv/ upon publication.
When available: With publication

Supporting Documents
Document types: None

Additional Information
Who can access the data: Data will be publicly available with a use agreement.
Types of analyses: For any purpose with restrictions on publishing from the data.
Mechanisms of data availability: Upon signed data use agreement.
